# User-relevant factors determining prosthesis choice in persons with major unilateral upper limb defects: A meta-synthesis of qualitative literature and focus group results

**DOI:** 10.1371/journal.pone.0234342

**Published:** 2020-06-30

**Authors:** Nienke Kerver, Sacha van Twillert, Bart Maas, Corry K. van der Sluis

**Affiliations:** 1 Department of Rehabilitation Medicine, University of Groningen, University Medical Center Groningen, Groningen, The Netherlands; 2 Centre of Expertise on Quality and Safety, University of Groningen, University Medical Center Groningen, Groningen, The Netherlands; University of Chicago, UNITED STATES

## Abstract

**Objective:**

Considering the high rejection rates of upper limb prostheses, it is important to determine which prosthesis fits best the needs of each user. The introduction of the multi-grip prostheses hands (MHP), which have functional advantages but are also more expensive, has made prosthesis selection even harder. Therefore, we aimed to identify user opinions on factors determining prosthesis choice of persons with major unilateral upper limb defects in order to facilitate a more optimal fit between user and prosthesis.

**Methods:**

A qualitative meta-synthesis using a ‘best-fit framework’ approach was performed by searching five databases (PROSPERO registration number: CRD42019126973). Studies were considered eligible if they contained qualitative content about adults with major unilateral upper limb defects experienced in using commercially available upper limb prostheses and focused on upper limb prosthesis users’ opinions. Results of the meta-synthesis were validated with end-users (n = 11) in a focus group.

**Results:**

Out of 6247 articles, 19 studies were included. An overview of six main themes (‘physical’, ‘activities and participation’, ‘mental’, ‘social’, ‘rehabilitation, cost and prosthetist services’ and ‘prosthesis related factors’) containing 86 subthemes that could affect prosthesis choice was created. Of these subthemes, 19 were added by the focus group. Important subthemes were ‘work/school’, ‘functionality’ and ‘reactions from public’. Opinions of MHP-users were scarce. MHPs were experienced as more dexterous and life-like but also as less robust and difficult to control.

**Conclusion:**

The huge number of factors that could determine upper limb prosthesis choice explains that preferences vary greatly. The created overview can be of great value to identify preferences and facilitate user-involvement in the selection process. Ultimately, this may contribute to a more successful match between user and prosthesis, resulting in a decrease of abandonment and increase of cost-effectiveness.

## Introduction

The absence of an upper limb, due to either an amputation or a congenital defect, can have a huge impact on a person’s life and can cause a decrease in life satisfaction [1]. Although the population with upper limb defects (ULD) is small compared to that with lower limb defects, people with ULD are typically young and have normal life expectancy [2,3]. Therefore, they are potentially long-term prosthesis users and consumers of prosthesis-related health care, resulting in high costs [4–6].

In the Netherlands, the yearly average cost per upper limb prosthetic user increased from €3070 in 2012 to €4130 in 2016 [7]. One of the likely reasons for the increased costs is the technological developments in the field of hand prostheses. New models of hand prostheses, like the multi-grip myoelectric hand prostheses (MHP) with moveable thumb and fingers, have been introduced to the market from 2006 onwards. The MHPs have functional advantages, but are more expensive, are generally more vulnerable and more difficult for users to control. For some people, a simpler prosthesis would probably meet their expectations and wishes equally well or possibly even better. Considering that the rejection rates for upper limb prosthesis are estimated as high as 35%, it is important to determine which prosthesis best fits the needs and wishes of each user [8].

Matching the user with the most suitable upper limb prosthesis is a challenge, and the introduction of MHPs has made this choice even harder. Another reason that seems to complicate prosthesis choice is that the goal of prosthesis use varies from person to person [8–16]. For some users the prosthesis might be essential to perform certain activities of daily living, while for others a natural appearance and social integration might be of greater importance [8–16]. Therefore, it is of utmost importance to clearly identify the individual expectations and wishes of the user during the prosthesis selection process. The current focus on shared decision-making in health care, especially for decisions involving preferences, confirms this need to understand the factors relevant for prosthesis choice from a user’s perspective [17,18].

Qualitative study approaches could lead to a better understanding of this user perspective. However, the literature on important prosthesis-related factors from a user’s perspective is diverse and fragmented. With ‘diverse’ we refer to the many different methods that are used in the current qualitative literature, like focus groups, interviews, open-ended questions in questionnaires, narratives, case-reports and commentaries [9,11,19–22]. By ‘fragmented’ we mean that various terms and constructs are used. For example, some studies focus on the user needs or goals, some on user preferences or satisfaction and others on concerns or prosthesis rejection [9,13,23,24]. Furthermore, most studies on persons with ULD report on a small number of participants. Thus, there is a gap in the literature reporting on opinions, needs and preferences of a large number of users regarding their prosthesis choice.

The qualitative findings in the diverse studies in the current literature can however supplement each other to create a more integrated picture of all factors that affect prosthesis choice. The meta-synthesis of qualitative literature from Murray and Forshaw et al. (2013) highlighted areas of importance for persons who underwent a limb amputation and started to use a prosthesis, such as particular coping strategies, identity issues, and both social and personal relationships [25]. However, their study did not focus on the process of prosthesis selection, and only two of the 15 studies included in their meta-synthesis focused specifically on persons with ULD. Therefore, we performed a meta-synthesis of qualitative literature focusing only on perspectives of persons with ULD. The ultimate aim of this study was to identify user opinions on factors that determine prosthesis choice of persons with major unilateral upper limb defects. Prosthesis choice was defined as a person’s choice to wear a particular type of prosthesis (a cosmetic, passive or body-powered prosthesis; a standard mono-grip myoelectric prosthesis (SHP); an MHP or a DEKA-arm) including the choice of not wearing a prosthesis at all. Although the DEKA-arm and MHP both have multiple grips, we considered these separately since the DEKA-arm is predominantly controlled by foot controls and the MHPs utilize standard myoelectric control. The results of the meta-synthesis were tested with end-users to assess whether they were correct and complete, in order to create a well-arranged overview of all factors that can determine prosthesis choice. This overview may help individuals with ULD to identify and prioritize preferences and needs in clinical practice, which can then be taken into account in the prosthesis selection process in order to establish a more optimal fit between the needs and wishes of the patient and best suitable upper limb prosthesis resulting in higher cost-effectiveness.

## Materials and methods

In part 1 of the study, a meta-synthesis of qualitative findings from literature was performed using a ‘best-fit’ framework (BFF) approach [26]. A BFF-approach is a pragmatic, transparent and efficient method for the synthesis of qualitative evidence, in which relevant literature is identified and used to create a preliminary framework with ‘a priori’ themes and subthemes [26]. Subsequently, the included data is coded against this preliminary framework and if data does not fit within the themes or subthemes of the preliminary framework, new inductive themes are added. So, with the BFF approach a preliminary framework is developed, tested and adjusted if necessary, resulting in the prefinal framework [26,27]. In part 2 of this study, the created prefinal framework was tested and completed by persons with ULD in a focus group meeting, resulting in the final framework.

### Part 1: Meta-synthesis of qualitative findings from literature

The meta-synthesis of qualitative findings from literature was registered in the International Prospective Register of Systematic Reviews’ (PROSPERO) with registration number: CRD42019126973 (https://www.crd.york.ac.uk/prospero/display_record.php?ID=CRD42019126973). The ‘enhancing transparency in reporting the synthesis of qualitative research’ (ENTREQ) approach was used as a reporting guideline for the meta-synthesis of the included studies [28].

#### 1.1 Development of the preliminary framework

According to Carroll et al. (2013) a BFF meta-synthesis requires the identification of relevant theories, conceptual models and relevant frameworks [26]. Therefore, and to make this meta-synthesis as reproducible as possible, the Behavior of Interest, Health context, Exclusions, and Models or Theories (BeHEMoTh) template and procedures were used to systematically identify relevant models and theories to create the preliminary framework [29]. First a BeHEMoTh question was formulated (Table 1). Since literature has shown that the user goals differ between persons with unilateral and bilateral ULD, and that a complete hand prosthesis can only be worn by persons with a level of ULD at or proximal from the wrist, we only focused on persons with such a major unilateral ULD [9]. Subsequently, the occurrence of theories and models was checked by searching internal and external databases. With ‘internal database’ the authors’ list of articles retrieved for the qualitative synthesis is meant (see section 1.2 of methods). The internal database was searched using the terms ‘theor*’, ‘concept*’, ‘framework*’ and ‘model*, which provided one suitable article [30]. In parallel, searches in external databases (PubMed, Cochrane Library, EMBASE, Cinahl and PsychInfo) were performed (S1 Text). A total of 822 studies were found, of which 746 were left after deduplication. The first reviewer (NK) screened the studies on title and abstract, which resulted in two possible suitable articles. After reading the full text, it turned out that both articles were not suitable, one because of its technical focus and one because it concerned a statistical model. Following the BeHEMoTH template, the next step was to combine the used models and theories in the found literature with the search terms used for either ‘Behavior of Interest’ or ‘Health Context’ [29]. In the single study that had been identified, the Anderson behavioral model of health utilization adapted to upper limb prosthesis acceptance and use, was presented. Therefore, the external databases were searched again using the terms ‘Anderson behavioral model of health utilization’, combined with either the search terms used for ‘Behavior of Interest’ or ‘Health Context’ from the BeHEMoTh template. In total 13 additional studies were found, which were screened on title and abstract by the first reviewer (NK). None of these studies were suitable for the development of the preliminary framework. All in all, after using the BeHEMoTh template and procedures, only one suitable study that presented a relevant theory, model or framework could be found. Therefore, both reviewers (NK, CvdS) suggested studies based on their knowledge of literature in the field, that did not present a model or theory, but had nearly identical study aims as formulated in the BeHEMoTh question although different methodologies were applied. One of the reviewers was a doctoral student in upper limb prosthetics (NK), and the other a rehabilitation doctor and professor specialized in upper limb prosthetics (CvdS). Two studies were suggested by the reviewers: one was a literature review about user needs of upper limb prosthesis users and the other was a modified Delphi study about important factors to consider in rehabilitation for persons with ULD [10,31].

**Table 1:** The Behavior of Interest, Health context, Exclusions, and Models or Theories (BeHEMoTh) question formulation for prosthesis choice behavior of persons with major unilateral upper limb defects (ULD).

Strategy	Terms
Be–Behavior of Interest	Prosthesis choice of persons with major unilateral ULD
H–Health Context	Home-situation or daily living
E–Exclusion	Non-theoretical, technical, statistical or economic model
MoTh–Models or Theories	Model, theory, framework or concept

To create the preliminary framework a list of factors that could affect prosthesis choice was retrieved based on the three included studies [10,31,32]. To further structure the list, factors were clustered in overarching themes. Six main themes were identified: ‘physical’, ‘activities and participation’, ‘mental’, ‘social’, ‘rehabilitation’ and ‘prosthesis related factors’. Within the main themes several sub-themes were identified (S1 Table).

#### 1.2 Search methods and study selection

For the identification of studies for the meta-synthesis of qualitative findings from literature the following electronic bibliographic databases were searched (search date: 26-04-2019): PubMed, Cochrane Library, EMBASE, Cinahl and PsychInfo. Together with a librarian specialized in search strategies for systematic reviews, search strings were composed using the SPIDER (Sample, Phenomenon of Interest, Design, Evaluation, Research type) strategy [33]. To keep the search string manageable and structured, we have chosen to divide it into two separate strings: one for persons with congenital ULD and one for persons with acquired ULD (S2 Text). Because MHPs are commercially available since 2006, we considered studies published between 2006 and 2019 in Dutch or English. The eligibility criteria for study selection can be found in Table 2.

**Table 2:** Eligibility criteria for study selection.

**Inclusion criteria**	**Clarifications**
• Qualitative content, reporting on adult participants with unilateral ULD at or proximal from the wrist.	• Qualitative content included any free-response questionnaire, interview text, focus group text, narrative or any other qualitative text.
• Study participants should have experience in using commercially available or Food and Drug Administration (FDA) approved upper limb prostheses in the home environment.	• To ensure that the results were as closely aligned as possible to clinical practice and reflect patient experiences of prosthesis usage in daily life.
• Focus on the opinions of upper limb prosthesis users about their prosthesis. This focus should at least be recognizable in the title or aim of the study.	• We searched for user opinions in a broad sense, since literature that only focuses on prosthesis choice was very limited.
**Exclusion criteria**	**Clarifications**
• The focus was on sport specific or 3D printed prostheses only.	• Exclusion if prosthesis were only used for very specific activities or were mostly experimental or research prostheses and were not intended for daily usage.
• > 25% of the participants did not fit the target population and in which the target population was not separately analyzed.	• To include sufficient text or quotes that mainly concerned the target population of this study.
• No full text was retrievable.	• Not possible to analyze study.
• It was a chapter from a book.	• Book chapters often include literature from published articles and were hard to retrieve.

The retrieved studies from all databases were merged and deduplicated. Thereafter, the studies were screened on title by the first reviewer (NK). If the first reviewer had any doubts about including the study based on title screening, these were included to the next round of screening. Subsequently, abstracts were independently screened on eligibility by two reviewers (NK, CvdS). Agreement between both reviewers was reached if both reviewers independently had the same judgment about including or excluding an article based on abstract screening. The agreement between both reviewers was calculated using Cohen’s kappa. Any disagreement between the reviewers was resolved through discussion until consensus was reached. After screening on title and abstract, the full text of the selected studies was read by the first reviewer to confirm inclusion criteria (NK). In case of any doubt, the second reviewer was consulted (CvdS). The reference sections of the included studies were checked for additional studies that were suitable for inclusion. Some of the included studies had a mixed methods design or also included participants who did not fit in the target population. In these cases only the relevant parts of the studies were included.

#### 1.3 Quality assessment

The Critical Appraisal Skills Programme (CASP) qualitative research checklist was used for quality assessment, which is a commonly used and user-friendly critical appraisal tool for qualitative research [34–36]. This checklist consists of ten questions that are divided into three parts: ‘Are the results of the study valid?’ (part A), ‘What are the results?’ (part B) and ‘Will the results help locally?’ (part C). The quality assessment was conducted independently by two reviewers (NK, BM). Any disagreement was resolved through discussion, if necessary a third reviewer was consulted (CvdS). Because the representation of the used methodology and reporting of a qualitative study is not an indicator for the overall value of the study, no studies were excluded based on the quality assessment [37].

#### 1.4 Data extraction and synthesis

The first reviewer (NK) extracted the following descriptive information from the included studies: participant demographics, study design, methods of participant recruitment and information needed to assess the risk of bias. For the qualitative data extraction of the main results all relevant text under the heading ‘results’ of the included studies was entered into Atlas.ti software. Text was considered relevant if it met the eligibility criteria of this meta-synthesis. Original quotes of participants as well as aggregated contributions on patient opinions provided by authors were included. For the analyses the main themes and subthemes of the developed preliminary framework were used. If a theme in the extracted data did not ‘fit’ within one of the existing ‘a priori’ themes or subthemes, a new theme was created and added to the framework. Subthemes that were not mentioned in the data at all, were deleted from the framework. The qualitative data analysis was performed independently by two reviewers (NK, BM). Any disagreements were resolved through discussion, consulting a third reviewer (SvT) were necessary. After the reviewers agreed with the adapted prefinal framework, all data was re-coded from blank for the final results (NK).

### Part 2: Focus group

The consolidated criteria for reporting qualitative studies (COREQ) checklist was used as a reporting guideline to ensure accurate and complete reporting of the focus group [38]. The local Medical Ethics Review Board of the University Medical Center Groningen (UMCG) judged that formal approval of the study was not needed (METc 2018/582). All participants provided a written informed consent.

A focus group was organized to test if the prefinal framework created with the meta-synthesis of qualitative findings in literature was correct and complete. The gained information of the focus group was merged with the prefinal framework into the final framework, which represents the most complete overview of factors influencing prosthesis choice for persons with major unilateral ULD. A focus group was chosen as a method since this is an appropriate method to test new concepts and get insight into personal and shared perspectives of people [38,39].

#### 2.1 Participants

Adult participants (over 18 years of age) with unilateral ULD at or proximal from the wrist that use an upper limb prosthesis, or had been using one in the past, were considered eligible. A purposive sample of potential participants from the UMCG, the Netherlands, including both males and females, current and former prosthesis users with different levels of upper limb defects, either acquired or congenital, were approached by letter. Furthermore, an advertisement for participation in the focus group was placed in the magazine of the patient organization for persons with an amputation or congenital limb defects in the Netherlands. All potential participants were sent an invitation with a detailed information letter and an informed consent form by post.

#### 2.2 Data collection

One focus group that took 75 minutes was organized in April 2019 in a meeting room at the UMCG. The focus group was moderated by a female expert in moderating focus group meetings (SvT). The assistant of the moderator was a female doctoral student in hand prosthetics (NK). Both, the moderator and assistant were not acquainted with the participants in advance. Two researchers, who were not involved in this study, were also present at the focus group meeting and helped with logistical matters. The focus group was audio-recorded and transcribed verbatim.

At the start of the focus group, a brief introduction into the subject and purposes of the meeting were given. Then, participants were asked two open questions: (1) which matters/factors influenced your prosthesis choice, or the choice to not use a prosthesis? (2) And after you had chosen a prosthesis (or to not use a prosthesis), which matters/factors were important for you when using a prosthesis? These open questions were asked without revealing the prefinal framework. Thereafter, participants were shown the themes and subthemes of the prefinal framework in a consecutive order, presented on large posters. By showing a new theme, participants were asked if they understood all the mentioned themes and subthemes, and if necessary explanations were given. Then participants were asked if any sub-themes of importance were missing, and if the overview contained subthemes that were unimportant to them (S3 Text). Participants were given enough time to respond on the shown theme and only after no additional comments were made by the participants, the next theme was introduced. At the end of the focus group, participants completed a short questionnaire about socio-demographic data (age, level of limb loss, origin of limb loss, current prosthesis, job).

#### 2.3 Data analysis

The transcript was entered into the Atlas.ti software, and a framework approach was used to analyze the data. Since the prefinal framework was already developed in the meta-synthesis of qualitative findings in literature, and we aimed to test the correctness and completeness of this framework, we chose to use the prefinal framework for the coding. New themes were added if the data did not ‘fit’ within the (sub-) themes of the prefinal framework. To ensure that we did not miss important aspects of the data, due to the mainly deductive approach, both analysts paid extra attention to the ‘fit’ of the data in the prefinal framework. New identified subthemes were added to the final framework. The qualitative data analysis was performed independently by two female coders, a doctoral student and a research assistant (NK, CB). Any disagreements were resolved through discussion. Data saturation was confirmed if both coders did not find new subthemes in the transcript of the focus group anymore. After the coders agreed with the final framework, all data was re-coded from blank for the final results (NK). The transcript and results of the focus group were not returned to the participants for comments or corrections. Illustrative quotes were translated to English (NK). The presented quotes could not be assigned to specific participants, since from the audiotapes identification of participants was not feasible.

## Results

### Part 1: Meta-synthesis of qualitative findings from literature

#### 1.1 Study selection

**Fig 1:**
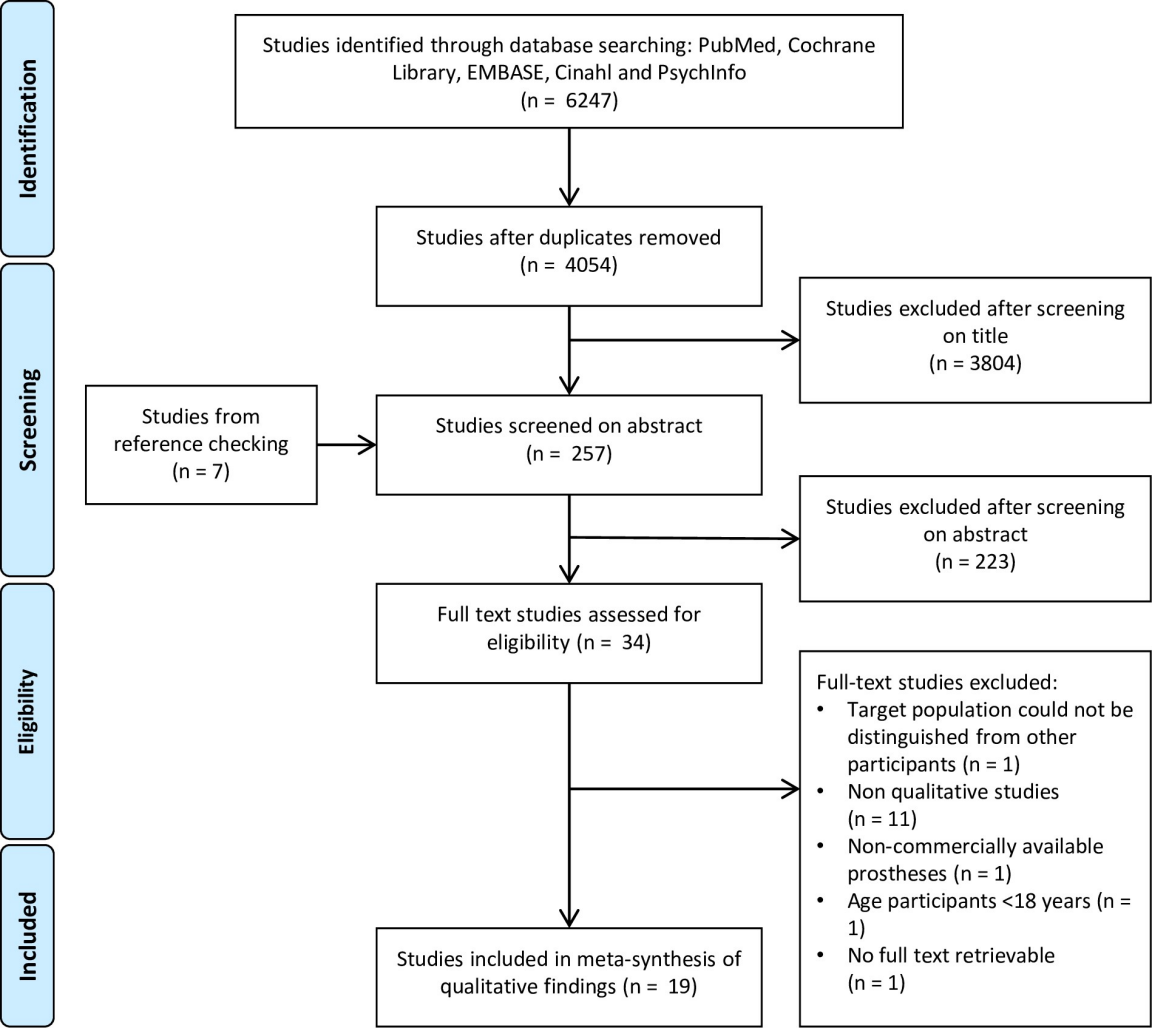
Preferred Reporting Items for Systematic Reviews and Meta-Analysis (PRISMA) flowchart for the selection of studies.

Out of a total of 6247 studies, 19 studies were included in the meta-synthesis (Fig 1; S2 Table). Seven of the included studies had a mixed methods design [9,12,19,23,40–42]; seven included participants of whom more than 25% did not fit the target population but in which the relevant population was analyzed separately [9,13,20,21,42–44]; and one contained data of using a prosthesis in both home and laboratory environment [45]. Of these studies, only the relevant parts were included. In three studies insufficient information was available to check if the included participants completely met the inclusion criteria [9,21,40]. However, since this was very likely the case, these were included. The agreement between both reviewers, calculated with Cohen’s kappa coefficient, was 0.60, which can be interpreted as a moderate level of agreement [46].

#### 1.2 Study characteristics

In total 479 participants from eight countries (United States, India, United Kingdom, Switzerland, Sweden, Netherlands, Canada and Italy) were included (Table 3). Study sample sizes varied between one and 145. Four studies only included men [19,23,42,47], and two only women [11,20]. In eight studies participants who did not fit the target population were included and not separately analyzed: three studies included participants younger than 18 years [12,40,44]; five studies included participants with bilateral upper limb loss [22,24,42,43,45]; two studies included participants with upper limb loss distal from the wrist [22,43]. In all of these studies, this involved less than 25% of the included participants.

**Table 3:** Summary of the sample characteristics, used methodologies and quality assessment from included studies.

Study	Sample size	Age	Gender	Origin of limb loss	Level of limb loss	Type of prosthesis	Wearing time prosthesis	Country (ISO-code)	Data collection technique	Data analysis	CASP criteria unmet ^A^
Zheng et al.(2019) [22] ^B^	11	Median: 45 years	9 M;	2 ULRD;	1 Wd;	Not reported	Not reported	USA	Focus groups and semi-structured phone interviews	Qualitative content analysis with an inductive approach	None
		Range: 27–65 years	2 F	9 AA	6 Tr;						
					1 Ed;						
					1 Th;						
					1 Bi Tr;						
					1 Dw						
Widehammar et al. (2018) [48]	13	62% ULRD (n = 8; median: 33 years; range: 20–47 years)	9 M;	8 ULRD;	10 Tr;	13 ME	6 Daily;	SWE	Semi-structured face-to-face or phone interviews	Qualitative content analysis with an inductive approach	3
			4 F	5 AA	3 Th		2 only at work;				
							3 only in specific situations;				
		38% AA (n = 5; median: 48 years; range: 27–74 years)									
							2 non-wearer				
Schweitzer et al. (2018) [19] ^B^	1	Not reported	1 M	1 AA	1 Tr	BP and MHP	10–12 hours a day	CHE	Case-report	Not applicable	Not applicable
Resnik et al. (2018) [11]	3	Median: 29 years	3 F	2 ULRD;	3 Tr	2 MHP, DEKA-arm and CP;	Not reported	USA	Semi-structured face-to-face interviews	Four of the authors compared each case to identify categories (called constant comparison) and applied the grounded theory approach	3, 4, 5 and 6
		Range: 24–32 years		1 AA							
						1 SHP, DEKA-arm and CP					
Davis & Onge (2017) [20] ^B^	1	46 years	1 F	1 ULRD	1 Tr	1 SHP	Not reported	USA	Commentary of prosthesis user	Not applicable	Not applicable
Benz et al. (2016) [24]	7	Range: 41–65 years	5 M;	1 ULRD;	5 Tr;	7 ME; (of whom 1 with ME and CP)	5 daily;	USA	Individual interviews	Step 1: initial coding, method not clearly mentioned	3, 4, 6, 8 and 9
			2 F	6 AA	2 Bi Tr		1 weekly;				
							1 monthly				
										Step 2: topic modeling using Latent Dirichlet Allocation (LDA), which is a form of machine learning	
Nagaraja et al. (2016) [40] ^B^	60	10% children (n = 6; mean: 6 years; SD 0.8 years);	54 M;	8 ULRD;	45 below elbow;	93% CP or BP;	Not reported	IND	Phone or face-to-face survey with open-ended questions	Not clearly mentioned	3, 5, 6, 8 and 9
			6 F ^C^	52 AA ^C^							
					15 above elbow ^**B**^	7% other^C^					
		85% male adults (n = 51; mean: 31 years; SD: 10.1 years);									
		5% female adults (n = 3; mean: 21 years; SD 5.3 years)									
Deijs et al. (2016) [41] ^B^	8	Mean: 50 years	6 M;	3 ULRD;	8 Tr	8 SHP	> 4 hours daily	NL	Semi-structured face-to-face interviews	Not clearly mentioned	6
		SD: 14 years	2 F	5 AA							
Luchetti et al. (2015) [23] ^B^	6	Median: 47 years	6 M	6 AA	6 Tr	6 SHP and MHP	> 8 hours daily	ITA	Clinical face-to-face interviews;	Qualitative content analysis using the ideographic case study approach of the Interpretative Phenomenological Analysis	3, 4, 5, 6 and 8
		Range: 35–65 years									
Wijk & Carlsson (2015) [49]	13	Mean: 43 years	5 M;	6 ULRD;	13 Tr	6 ME;	13 daily users, of whom 7 wear prosthesis whole day	SWE	Semi-structured face-to-face interviews	Qualitative content analysis	6
		Range: 29–71 years	8 F	7 AA		5 CP;					
						2 both					
Resnik et al. (2014) [45] ^D^^,^ ^E^	37	64.9% Gen 2 (n = 24; mean: 44.4 years; SD: 16.9 years)	32 M;	Not reported	9 Tr;	All DEKA-arm + other prosthesis (unknown which type)	18 full-time;	USA	Face-to-face survey with open-ended questions	A qualitative approach to content analysis, followed by a cross-case analysis to compare the users’ perspectives	3, 5 and 6
			5 F		9 Th;		14 part-time;				
					10 Shd;						
		35.1% Gen 3 (n = 13; mean: 46.4 years; SD: 16.4 years)			4 Bi						
							5 non-wearer				
Horst & Hoogsteyns et al. (2014) [21] ^B^	7	Not reported	Not reported	Not reported	Not reported	Not reported	Not reported	NL	Biographical face-to-face interviews	Biographic Narrative Interpretation Method	1, 4, 7 and 9
Vasluian et al. (2013) [44] ^B^	12	Range: 17–20 years	4 M;	12 ULRD	12 Tr	3 ME;	1.5–12 hours daily; 7 non-wearer	NL	Online focus group interviews	Framework approach	None
			8 F			2 CP;					
						7 none					
Waldera et al. (2013) [43] ^B^	17	Only the age at amputation was reported:	Not reported	17 AA	1 Wd;	Not reported	11 Wearer;	USA	Focus group and face-to-face or phone interviews	Inductive thematic analysis	8
					7 Tr;		6 non- wearer				
					3 Th;						
		<20 years: 17.6%			2 Shd;						
		20–29 years: 23.5%			3 Bi;						
		30–39 years: 17.6%			1 Dw						
		40–49 years: 5.9%									
		50–59 years: 5.9%									
		≥ 60 years: 5.9%									
		Unknown: 23.5%									
Bouffard et al.(2012)	12	Mean: 56.6 years	12 M	12 AA	11 Tr;	1 ME;	3 for 2–6 hours daily;	CAN	Focus group meeting and face-to-face semi-structured interviews	Thematic analysis approach	5
		SD: 16.5 years			2 Th (of whom 1 Bi)	8 BP;					
[42] ^B^						3 both	9 more than 6 hours daily				
Schaffalitzky et al. (2009) [13] ^B^	2	Not reported	1 M;	1 ULRD;	1 Tr; 1 Th	1 SHP;	1 for 4 hours a day for 4 days a week;	USA	Face-to-face interviews using the RGT	‘Contrast Method’ or triadic elicitation, which is a method that’s used in all RGT studies to generate constructs on which to rate elements	4 and 5
			1 F	1 AA		1BP					
							1 for 18 hours a day for 7 days				
Saradjian et al. (2008) [47]	11	Median: 54 years	11 M	11 AA	1 Wd;	Not reported	At least weekly	UK	Semi-structured face-to-face interviews	Interpretative Phenomenological Analysis	7
		Range:			5 below elbow;						
		31–64 years									
					4 above elbow;						
					1 Shd						
Kyberd et al. (2007) [12] ^B^	113	16–20 years: 9%	68 M;	Not reported	65 Wrist;	30 ME;	76% for > 8 hours daily, of whom 46% for > 12 hours daily;	SWE, UK	Postal questionnaire with open-ended questions	Not clearly mentioned	1, 3, 6 and 7
		21–30 years: 12%	40 F;		35 Elbow;	68 CP;					
		31–40 years: 20%	5 missing responses		8 Shoulder;	15 other					
		41–50 years: 13%			5 missing responses						
		51–60 years: 22%									
		61–70 years: 16%									
							9% only occasional; others not reported				
		70–80 years: 4%									
		81+ years: 3%									
		Missing responses: 3%									
Biddiss et al. (2007) [9] ^B^	145	Mean: 43 years	Not reported ^F^	41% ULRD	Not reported ^F^	81 ME;	Not reported	CAN, USA, NL	Online or paper questionnaire with open-ended questions	Not clearly mentioned	6
		SD: 15 years				58 BP;					
						38 CP;					
						11 other					

^**A**^ CASP criteria: (1) Was there a clear statement of the aims of the research?; (2) Is a qualitative methodology appropriate?; (3) Was the research design appropriate to address the aims of the research; (4) Was the recruitment strategy appropriate to the aims of the research; (5) Was the data collected in a way that addressed the research issue?; (6) Has the relationship between researcher and participants been adequately considered?; (7) Have ethical issues been taken into consideration?; (8) Was the data analysis sufficiently rigorous?; (9) Is there a clear statement of findings?; (10) How valuable is the research?

^**B**^ Only qualitative parts of the study about experiences of the target population with commercially available prostheses were included, other parts of the study were not included in this overview.

^**C**^ The pediatric group was not analyzed separately, so this group (n = 6) is included in the results shown here.

^**D**^ Only the part of the study where the DEKA arm was compared with their current prosthetic device was included in the meta-synthesis.

^**E**^ This study included persons fitted with the Gen 2 and Gen 3 DEKA arm, 5 participants were included in both parts (Gen 2 and Gen 3)

^**F**^ Not reported separately for the relevant population of this review.

ISO-code, country code assigned by the International Organization for Standards; CASP, The Critical Appraisal Skills Programme qualitative research checklist; SD, standard deviation; ULRD, upper limb reduction deficiency; AA, acquired amputation; M, male; F, female; Tr, transradial; Th, transhumeral; Shd, shoulder disarticulation; Ed, elbow disarticulation; Wd, wrist disarticulation; Bi, bilateral; Dw, distal from wrist; SHP, standard myoelectric hand prosthesis (with only one grip function); MHP, multi-grip myoelectric hand prosthesis; ME, myoelectric (unknown which subtype); BP, body-powered prosthesis; CP, cosmetic/passive prosthesis; RGT, repertory grid technique; USA, United States; IND, India; UK, United Kingdom; CHE, Switzerland; SWE, Sweden; NL, Netherlands; CAN, Canada; ITA, Italy.

Most used data collection techniques were semi-structured interviews (conducted by phone or face-to-face), focus groups and questionnaires with open-ended questions. Furthermore, a case-report and a commentary were included [19,20]. Data analysis methods mainly concerned qualitative content analysis and thematic analysis (Table 3).

#### 1.3 Quality assessment

CASP criteria are presented if they were unmet, which was the case when a question was answered with ‘no’. Two of the included studies did not have any unmet CASP criteria [22,44], seven studies had one unmet criterion [9,41–43,47–49], one study had two unmet criteria [13] and seven studies had three or more unmet criteria [11,12,21,23,24,40,45]. Two studies could not be assessed with the CASP, since one was a case report and the other a commentary [19,20].

#### 1.4 Findings of meta-synthesis

The meta-synthesis of literature resulted in six main themes containing 65 subthemes (S1 Table). The main theme ‘rehabilitation’ did not cover all the contents of the included data within this theme. Therefore, this main theme was changed into: ‘Rehabilitation, costs and prosthetist services’. Below the results of most quoted subthemes are described.

*Theme 1*: *Physical*. The theme ‘physical’ included all subthemes that were directly related to the body of a person (e.g. physiological functions of the body, gender, age). Reasons to choose a prosthesis in relation to physical problems were often related to ‘overuse symptoms’: to relief already existing overuse complaints or to prevent getting overuse complaints [9,19,20,23,24,41,44,47–49]. Contradictory opinions on MHPs with regard to overuse complaints were found: in one study participants using an MHP experienced more relief of shoulder burden compared to an SHP, while in another the participant experienced more overuse complaints with an MHP in comparison to a body-powered prosthesis [19,23]. Next to the different types of prostheses involved in both studies, these contradictory opinions could be explained by the differences in employment, since the participant in the study of Schweitzer et al. (2018) had a physically demanding work environment and the participants of the study of Luchetti et al. (2015) did not [19,23].

‘I do it [activity of daily living] in a more natural manner.... The movements are normal movements.... I do not block the wrist because I can do like this [leans prosthetic hand on leg]. Now, the shoulder is in a normal position.’–Quote of a current MHP-user, with experience in using an SHP [23].

Another recurrent subtheme in the data was the relation between ‘phantom limb pain/sensations' and prosthesis choice [23,42,43,49]. Some persons indicated that phantom limb pain increased when wearing a prosthesis [42,49]. However, this was not always the case [23,42,49]. In one study it was suggested that interactions, both positive and negative, between the prosthesis and phantom limb pain/sensations was more common in myoelectric compared to body-powered prostheses [42].

*Theme 2*: *Activities and participation*. The theme ‘Activities and participation’ included all subthemes that referred to tasks and activities executed by a person as well as subthemes that referred to a person’s involvement in life situations. The subtheme ‘work/school’ appeared frequently in the data. The majority of the persons wore their prosthesis in the occupational/school setting, it enabled them to perform more ‘work/school’ related activities [9,11,19,20,23,24,42,44,47,49]. Myoelectric prostheses in general seemed to be less suitable for physically demanding work because they appeared to break easily and were not water- and dirt resistant [19,43]. Specifically regarding MHPs experiences differed, which seems to be person- and job-dependent: one participant said that the possibility to perform a lateral grip allowed him to do more work-related activities [23], while another person said he preferred the body-powered prosthesis over the MHP for his also physically demanding work, because he experienced the body-powered prosthesis as more reliable, comfortable, powerful, light-weight and cost-effective service with less maintenance [19]. In contrast, one DEKA-arm user, who also had an MHP, preferred to wear the MHP at work because of the more realistic look [11].

‘… it looks realistic and for me, at work, having something that looks realistic is crucial, so I couldn’t really wear the DEKA Arm.’–Quote of a DEKA-arm user, who also uses an MHP [11].

Many persons experienced their prosthesis useful for ‘leisure activities’ [9,23,24,41,44,47–49], although some persons could not perform all leisure activities they liked with their prosthesis [21,47,48]. As a consequence, they often did not enjoy those activities anymore and avoided them.

A part of the MHP-users experienced ‘grabbing, picking up and holding objects’ as more natural and more precisely using an MHP compared to an SHP [23]. However, one person experienced more frequent drops and skin problems when grabbing or lifting objects with an MHP compared to a body-powered prosthesis [19]. So the experiences with the MHP differ across persons, which seem to be person- and job dependent.

*Theme 3*: *Mental*. The theme mental included all subthemes that were related to someone’s thinking or feelings. ‘Coping’ strategies varied across people and could influence prosthesis choice and usage in both a positive and negative way [11,13,20–24,43,47,48]. For example, one participant said she felt miserable when the socket got sweaty, while another was not bothered by sweatiness, illustrating different types of coping styles [48]. Additionally, data emphasized that a prosthesis should fit someone’s ‘self-image’ or identity [11,20,21,23,47–49].

‘… There were some days at school when I was younger, when I couldn’t use the prosthesis and it was terrible… ‘–Quote of a prosthesis user, unknown which type [49].

Whereas some persons saw their prosthesis more as a clothing or fashion item, others saw their prosthesis as a part of their body and did not feel ‘complete’ without it [23,24,45,47–49]. This ‘embodiment’ was described by persons using an MHP [23], as well as by persons using an SHP and unknown prosthesis types [23,24,47–49]. The prosthesis seemed to have a positive influence on self-confidence [13,21,23,44,47,49]. With regard to MHPs, the influence seemed to differ per person: one participant felt more confident wearing an MHP because it felt more human, while another did not feel confident at all with an MHP [23]. This lack of confidence was probably caused by temporary failure of the MHP.

*Theme 4*: *Social*. The theme ‘social’ included all subthemes that relate to someone’s social relations and functioning in society. ‘Fitting in’ appeared to be a dominant subtheme [9,11,49,13,20,22,23,40,44,47,48]. People often had the desire to fit in and feel like everybody else. ‘Fitting in’ seemed to be closely related to the subtheme ‘reactions in public’ [9,11,20,23,40,44,47–49]. Most persons with ULD gain unwanted attention by people staring at their prosthesis or amputated arm. For some persons this was the reason to choose for a cosmetic prosthesis [49], but also for persons with other types of prostheses this often determined their prosthesis choice [9,11,20,23,40,44,47–49]. Participants with an MHP felt more normal with their prosthesis, because they could assume more natural postures and move more natural [23]. Furthermore, the MHP may facilitate social integration [23].

‘I was at a buffet… and, a glass of wine… I held a stem glass [with the Michelangelo prosthetic hand]!… Before, I had to take a plate from the buffet and find a table to eat, not now! Now, I can also walk… I mean… the fact that I have to sit at a table, it was something that isolated me from others.... Instead, now it is normal… wonderful!’–Quote of a current MHP-user, with experience in using an SHP [23].

*Theme 5*: *Rehabilitation*, *costs and prosthetist services*. The theme ‘rehabilitation, costs and prosthetist services’ included all subthemes that were directly related to training, guidance, prosthesis maintenance, reimbursement procedures and other services provided by either the rehabilitation team or the prosthetist. Most quoted subtheme in relation to this main theme was ‘professional maintenance of prosthesis’ [9,11,19,40,42–44,47]. The frequency in which professional maintenance was needed was experienced as a disadvantage of prostheses. No differences between prosthesis types were mentioned. The ‘costs of a new prosthesis and repairs’ were experienced high by most participants [9,19,40,43]. As a consequence, not all prosthesis options were financially accessible for everyone [9]. Possibly, users of a body-powered prosthesis considered costs more important than users of cosmetic or myoelectric prostheses [9].

‘The overall cost of the prosthesis needs to be lower. Most “insurances” pay 80%, but the other 20% is brutal.’–Quote of a prosthesis user, unknown which type [9].

‘A number of the farmers interviewed reported traveling long distances to see a prosthetist with the expertise they needed, which further increased their out-of-pocket cost. Some drove over 3 h one way, some traveled out of state, and some even traveled by air to see their prosthetist!’–Text from included study [43].

*Theme 6*: *Prosthesis related factors*. The theme ‘prosthesis related factors’ included all subthemes that were directly related to (properties of) the prosthesis. The overall most quoted subtheme was ‘functionality’ [9,11,41–45,47–49,12,13,19,20,22–24,40]. Repeatedly, it was mentioned that the prosthesis should be ‘functional’ and should have an added value relative to not having a prosthesis. For some this added value was indeed the function of the prosthesis, while for others a life-like appearance was more important. For some a prosthesis did not have any added value and they therefore chose to not use one [9,11,49,13,20,21,23,44,45,47,48]. Most MHP-users indicated that the prosthesis had more functions and resulted in an increased dexterity [23]. However, users also indicated the device was less robust and noisier [23].

The importance of a ‘life-like appearance’ of the prosthesis for persons differed [9,11,41,44,47–49,12,13,20–24,40]. For some persons a life like appearance did not matter at all, while for others this was the main reason to choose a particular prosthesis.

‘You’re very conscious of the fact that…I mean, me and my wife don’t find it off-putting but I think other people would. I won’t even wear a short-sleeved shirt because I’ve only got one arm showing and I’m obviously disabled.’–Quote of a prosthesis user, unknown which type [47].

A recurrent complaint about the appearance of the prosthesis, especially from females, was the relatively big size of some prosthetic hands [9,11,12,23,24,48]. Due to the size of the hand, and also the socket, not all clothes could be worn. Sometimes the prosthesis was too big to get into a sleeve. With regard to MHPs, it was mentioned that the MHP was slightly bigger compared to SHPs [23]. However, despite this difference, users said the MHP looked more life-like [23].

‘Wearing comfort’ was frequently experienced as a drawback of prostheses [9,11,41,42,44,47–49,12,13,19,20,22–24,40]. For body-powered prosthesis users, the harness was the main cause of discomfort, while for myoelectric prosthesis users the socket was [9,20,48]. In case of myoelectric prostheses, some persons said this was due to a poor socket fit in combination with sweating, that could cause slipping off the prosthesis. Cold weather was also designated as a cause of discomfort, since the socket of the myoelectric prosthesis conducts the cold. A part of the MHP wearers indicated pain or tiredness of the stump due to friction and the heavy weight of MHPs [19,23].

‘Reliability of a prosthesis’ was of great importance for most users [9,11,44,47–49,12,13,19,20,23,40,41,43]. The experienced reliability varied across different types of prostheses. While cosmetic prostheses were experienced as reliable [13], this was not always the case for MHPs, where unexpected movements could occur or an intention to move resulted in no movement [19,23]. ‘Durability’ was also seen as a major drawback of the current prostheses [9,12,49,13,19,20,22,41,43,45,47]. Durability of the body-powered prosthesis was experienced as better compared to the myoelectric [20,43]. For some persons, this was the main reason to not use a myoelectric prosthesis.

‘Control cables and wrists were identified as the “weak links” in body-powered upper-limb prostheses. Concern for durability was the main reason farmers with amputations gave for not utilizing myoelectric devices.’–Text of included study [43].

### Part 2: Focus group

#### 2.1 Participant characteristics

A total of 22 eligible participants were approached by letter, of whom 11 responded and agreed to participate (Table 4). Additionally, four persons responded to an advertisement in the magazine of the patient association for persons with an amputation or congenital limb defect. However, none of these four were able to participate in the focus group study due to excessive travel distances or being unavailable at the time the focus group was scheduled. Median age of the participants was 46.3 years (range: 31.4–69.7 years). Two of the 11 participants did not use a prosthesis (18.2%).

**Table 4:** Characteristics of the 11 participants of the focus group.

Characteristics	n (%)
**Gender**	
Male	6 (54.5)
Female	5 (45.5)
**Side of ULD**	
Left	8 (72.7)
Right	3 (27.3)
**Origin of ULD**	
Congenital	6 (54.5)
Acquired amputation	5 (45.5)
**Level of ULD**	
Transhumeral	4 (36.4)
Transradial	3 (27.3)
Wrist disarticulation	4 (36.4)
**Type of current prosthesis**	
SHP	4 (36.4)*
MHP	4 (36.4)*
Cosmetic/passive	2 (18.2)
None	2 (18.2)
**Experience with current prosthesis**	
< 1 year	2 (18.2)
1–5 years	4 (36.4)
> 5 years	3 (27.3)
Not applicable	2 (18.2)
**Work**	
Physically demanding	1 (9.1)
Mentally demanding	3 (27.3)
Physically and mentally demanding	3 (27.3)
Unemployed	3 (27.3)
Retired	1 (9.1)

ULD, upper limb defect; MHP, multi-articulating myoelectric hand prosthesis; SHP, Standard myoelectric hand prosthesis (with only one grip function).

^*^One participant had both an MHP and an SHP.

#### 2.2 Findings of focus group

**Fig 2:**
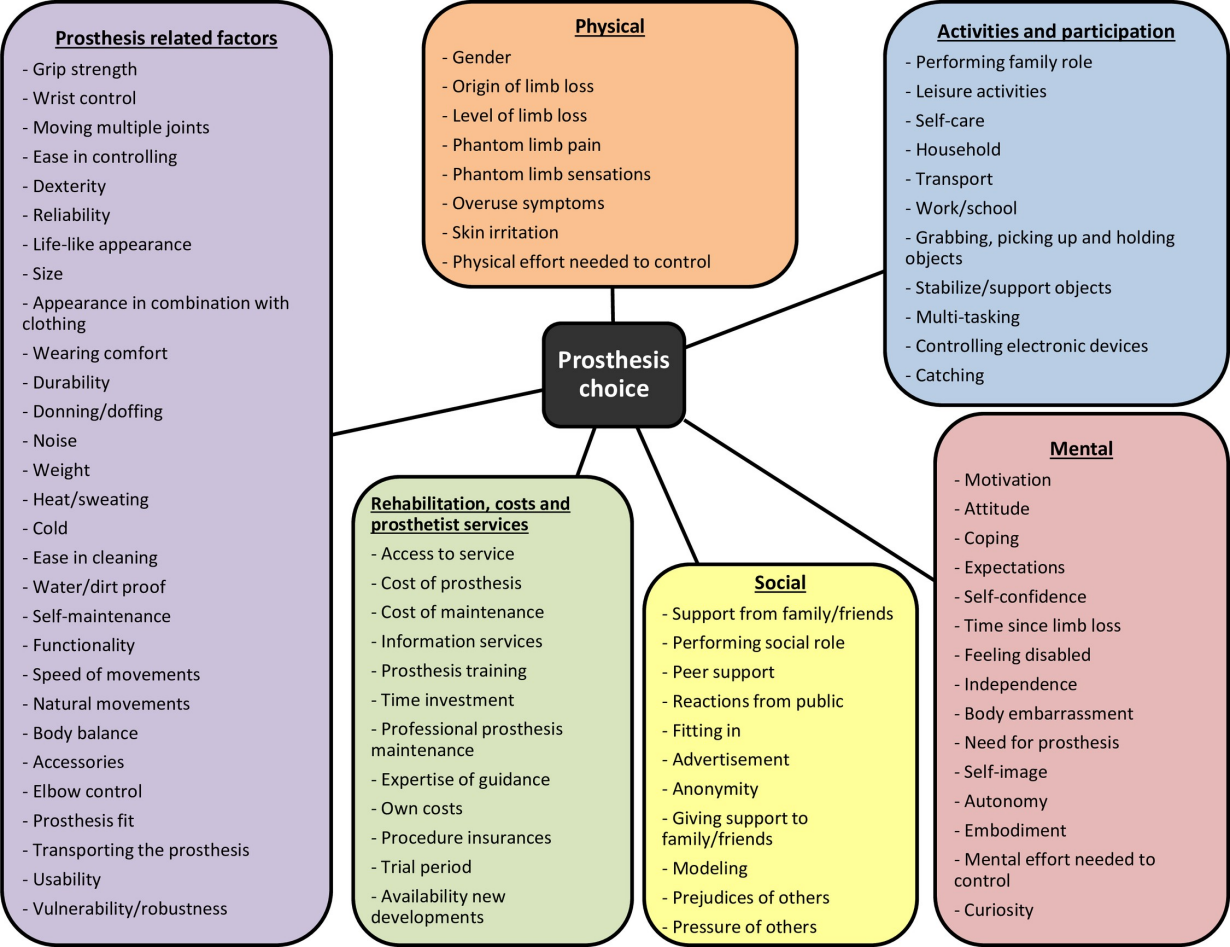
Final framework of factors determining prosthesis choice of persons with major unilateral upper limb defects.

The opinions of the participants supported the six main themes of the prefinal framework (Fig 2). A total of 19 additional subthemes were suggested by the focus group participants and were added to the framework. Furthermore, two subthemes were split based on the data of the focus group (S1 Table). Below the changes to the framework are discussed per main theme. If a subtheme is not discussed below, all participants agreed about the subtheme, which was then included in the final framework.

*Theme 1*: *Physical*. The participants emphasized that phantom limb pain and phantom limb sensations should be regarded as substantially different. While phantom limb sensations were experienced as something positive, phantom limb pain was experienced as negative. Phantom limb pain and sensations were therefore split into two separate subthemes in the final framework. Furthermore, participants indicated that the origin of limb loss was important with regard to choosing a prosthesis. Persons with congenital ULD were used to their situation, and for instance took their disabilities into account when choosing a job. In contrast, persons with an acquired amputation were used to do things with two hands, and were more in need of a prosthesis to learn everything all over again after the amputation. Therefore, the subtheme ‘origin of limb loss’ was added to the framework.

*Theme 2*: *Activities and participation*. The subtheme ‘catching’, like catching a ball or keys that were thrown, was suggested by one of the participants. This activity was not yet covered by the other themes, and therefore added to the final framework. Participants emphasized that during the selection process it is important to consider the activities you aim to do with a prosthesis. In particular, employment was often a motivation to choose for a particular prosthesis. But this worked the other way around as well: career choices, especially for persons with congenital ULD, were often dependent on prosthesis options.

‘Anyway, it is … important to, … if you have to choose [a prosthesis], if you have a congenital defect, to take into account certain professions you may want to do… what possibilities you have in that profession and what limitations possibly can be solved [by a prosthesis]?’

Some participants indicated that they preferred a myoelectric prosthesis because they needed to be ‘functional’ at their job. With regard to ‘transport’, one participant indicated she chose for an SHP instead of an MHP because when using the SHP she could make faster movements and the SHP came with a flexible wrist, which enabled her to drive a car and ride a bike more easily.

*Theme 3*: *Mental*. When participants were asked about the factors that influenced prosthesis choice, ‘curiosity’ was the first thing they answered. One person explained that she was curious about how life would be with a prosthesis and if she would be able to do more. Therefore, this subtheme was added to the framework. Furthermore, the focus group emphasized that the ‘need’ for a prosthesis was decisive whether to choose or not to choose for a prosthesis. For this reason, some persons wore their prosthesis only outdoors.

‘what she also says, I have no interest in a prosthesis, that’s also the reason that I immediately take it off at home…… and on, at work too, that if I have activities where I don’t need it then I take it off and the colleagues know that too… they don’t care’

*Theme 4*: *Social*. Six subthemes were added to the final framework: ‘anonymity’, ‘prejudices of others’, ‘modeling’, ‘pressure of others’, ‘advertisement’ and ‘giving support to family/friends’. A recurring topic in the focus group was the attention they gain from the public about their amputation and/or prosthesis. For some participants this was also the main reason to choose for a particular prosthesis, while others were not concerned about the reactions of others. As the quote below indicates some persons like to be a bit more anonymous in public.

‘… if I am just at home, I just take it [the prosthesis] off, but for me that was indeed very important, how the environment reacts, that they don’t immediately look at you, you still want to be a little bit anonymous in the environment… At least, that was my motivation for the prosthesis’

Regularly, participants were confronted with the prejudices of others, which often annoyed them. However, sometimes the prosthesis or short arm could trigger something in others that these persons could learn from, also described as ‘modeling’. This was experienced as something positive. Furthermore, others could sometimes exert pressure on the participants. One participant gave as an example that she never felt the need for a prosthesis, but because one of her clients kept on asking about it, she got curious and felt the pressure to try it.

‘… I did volunteer work with an old lady and every week she asked: “well, do you take a prosthesis?” And then I thought ehh… No… But she asked it so often that I thought, now I want to know what it is like [to have a prosthesis]’

Sometimes this pressure could also be caused by advertisements. When friends/family had seen a new video about a ‘super’ hand prosthesis, they received a lot of messages and some experienced that as pressure as well. Furthermore, some participants with congenital ULD indicated that their parents in particular had a hard time processing the fact that they had a short arm. So in addition to getting support from friends/family, giving support to family/friends was also important.

*Theme 5*: *Rehabilitation*, *costs and prosthetist services*. Five additional subthemes were suggested by the participants and added to the final framework: ‘trial period’, ‘expertise of professionals’, ‘own costs’, ‘procedure insurances’, and ‘availability of new developments’. In the Dutch health care system potential upper limb prosthesis users can try different prosthesis types at home for short periods, before they make their definite selection. Participants found this very helpful in choosing which prosthesis would suit them most.

‘I had three arms, that I could try [at home]…. to see how everything went, and then I could go home for two weeks, I could practice…. Yes, I liked that’

In addition, one participant indicated that the expertise of clinicians was also important when going through this selection process. The total costs, including the cost of the prosthesis and of maintenance, were experienced as high, especially if the person did not have other health care costs and had to pay this from the deductible.

‘… If you order it [new gloves], you have to deduct it from your own risk to reimburse… yes, that will cost you €250 again… if you have no further healthcare costs then that is a shame actually.’

The procedures of getting approval from the health insurance was also experienced as cumbersome. For example, the applications for compensations by the health care insurer were not always approved and could cause extra paperwork and delays.

‘… you have been doing without a prosthesis for 30 years, why do you suddenly need it? … Yes, and that should be well-argued [to get reimbursement from health insurances]…’

Lastly, the availability of new models of hand prostheses in clinical practice was too slow according to a few participants. They had for instance tried a new model hand for research goals and could not get one in clinical practice yet.

Focussing on MHPs, two aspects within this theme were highlighted. Prosthesis training sessions could only be done at the hospital. One person experienced this as the main disadvantage of the MHP. Another participant explained that the MHPs needed relatively more repairs, and that repairs often took a few weeks or even more, which was the reason for this participant to choose for an SHP.

*Theme 6*: *Prosthesis related factors*. Seven subthemes were added to the framework: ‘usability’, ‘vulnerability/robustness’, ‘elbow control’, ‘prosthesis fit’, ‘accessories’ and ‘transporting the prosthesis’. The usability of prostheses was important for the majority of the participants. If a prosthesis was difficult to use, and if this did not outweigh the advantages, then the participants indicated they would rather not use a prosthesis. Additionally, the ease of controlling a prosthesis was indicated as important by a part of the participants. One participant chose consciously for an SHP instead of an MHP, because of the difficulties in controlling an MHP. Participants who used an MHP, confirmed that they sometimes experienced difficulties in control, especially in the beginning.

‘In the beginning, with the I-limb [an MHP], I could close it well, but sometimes I couldn’t open it, but everyone wants to shake hands with you when you are wearing it [the new MHP]. But yes, than you’re standing there, and you can’t open it [the MHP] and that’s just super uncomfortable’

Another subtheme that emerged was the vulnerability or robustness of a prosthesis. Participants agreed that a prosthesis should be ‘strong and robust’. Some prostheses were more vulnerable than others. For one participant this was the main reason to choose for an SHP.

‘… and because others, eh, the advanced ones, like the I-limb [an MHP], are at risk to malfunction, …’

Participants noted that ‘wrist control’ was included in the framework, but ‘elbow control’ was not. For persons with more proximal levels of limb absence elbow control could also be important. So this subtheme was added to the final framework. Furthermore, participants of the focus group mainly talked about symmetry and balance of the body, while the meta-syntheses mainly emphasized the way of moving with the prosthesis, and whether these movements were life-like. As these themes seem to reflect two different matters the subtheme ‘natural movements/postures’ was split.

## Discussion

The aim of the study was to identify user opinions on factors determining prosthesis choice of persons with major unilateral ULD. An overview of 86 factors that could affect prosthesis choice was created. A person with ULD cannot possibly know all factors that could be decisive for prosthesis choice. So if a clinician asks a potential prosthesis user which factors they think might be of importance, it is highly unlikely that they will mention all factors that really matter, simply because the potential user does not know that these factors can matter. Our extensive overview can be used by professionals to provide potential prosthesis users more insight into all factors that may matter. That way the process of shared decision-making can be facilitated and subsequently the conversation on factors most important to that individual person can start.

Regarding prosthesis choice in relation to the ‘new’ MHPs, results show that the MHP may improve social integration and was experienced as more dexterous and more life-like compared to other prostheses. However, disadvantages of the MHP were also addressed: less robust, less durable, relatively big size, difficult to control, noisier and cause of stump complaints. It should however be noted that the meta-synthesis and focus group included only nine and four MHP-users, respectively. As a consequence, the final framework may still be lacking factors related to the choice of an MHP. In the light of the high costs related to MHPs, it is striking that only very few studies have been published about user experiences with MHPs. We therefore would encourage research into MHPs, especially studies focusing on determining for whom an MHP would be preferable above less expensive prostheses.

Results of this study suggest that MHPs seem to be less durable than other prostheses. If MHPs indeed need more repairs and maintenance, these costs are incurred in addition to the price to purchase the MHP. Blough et al. (2010) compared the estimated five-year cost of prosthetic and assistive devices between Vietnam and Operation Iraqi Freedom (OIF) veterans with limb loss [6]. These costs were $31,129 and $117,440 for both groups of veterans with upper limb loss, respectively. The OIF group used relatively more prostheses and more advanced types of prostheses than the Vietnam group. This observation implies that the introduction of more advanced types of hand prostheses indeed resulted in an increase of costs. However, cost-effectiveness of upper limb prostheses has not been thoroughly investigated [50]. To allow making evidence-based and cost-effective decisions regarding prosthesis choice, studies that compare the functionality, satisfaction and cost-effectiveness of upper limb prosthesis users are needed.

Needs of upper limb prosthesis users differ per person, as has also been noted by Carey et al. (2015), who performed a review on the differences between myoelectric and body-powered prostheses [51]. In their review the evidence was insufficient to conclude that the myoelectric or body-powered prostheses offered general advantages over the other. Therefore, they recommended that prosthesis choice should be based on individual user needs. Our results agree with their recommendation, but provide additional insights into user opinions and factors that could contribute to the prosthesis selection process.

Some limitations of this study should be taken into account. First, there were too few studies reporting on theoretical models in the literature that could be used for the preliminary framework. Therefore, two additional studies were selected by the reviewers. However, since the best-fit method also includes an inductive analysis of data, the effect of this bias will probably be very limited. Second, different study types were merged in the meta-synthesis: face-to-face interviews as well as open questions derived from questionnaires. Consequently, sometimes the opinion of a group was revealed, while at other times the opinion of only one person was presented. Third, in eight of the studies included in the meta-synthesis participants only partly fitted the target population [12,22,24,40,42–45]. In all studies this accounted for less than 25% of the participants, but it may have affected the results. Fourth, the focus of a few of the included studies was very specific. For instance, one study included only participants with phantom limb sensations [42]. However, with regard to these three last limitations the different types of studies enriched each other by emphasizing different aspects that could be of importance in the selection of a prosthesis. Fifth, since this study focused on prosthesis choice from the perspective of upper limb prosthesis users themselves, it would have been preferable to only include original quotes from such individuals. However, only providing original quotes would have led to a substantial loss of information, since quotes were mostly used to illustrate aggregated contributions on patient opinions provided by authors of the included studies. Therefore, we decided to include quotes as well as aggregated text provided by authors. Last, we could not assign quotes of the focus group to specific participants. Therefore we could not identify which quotes were related to MHP-users, unless explicitly mentioned by the participant themselves. For future research, we would suggest to videotape focus group sessions to prevent such problems.

An important strength of the study was the validation of our preliminary framework with the opinions of persons with major unilateral ULD. The input of the focus group was substantial and valuable, which confirmed that involving the target group is a prerequisite for the creation of user-relevant frameworks. Furthermore, the meta-synthesis of qualitative findings and the focus group both are very suitable to give an in-depth understanding of user opinions [28,52]. Another strength of this study was the systematic and structured approach to set-up and report of both the meta-synthesis and focus group.

The focus group included a diverse group of participants with regard to age and gender, origin and level of limb loss, prosthesis wearers/non-wearers and type of prosthesis (cosmetic/SHP/MHP). In addition, a group of 11 people was found to be sufficient to reach data saturation. However, it is possible that another group, for example, of another nationality or cultural background, would propose other factors. The accessibility of health care, health care systems, costs, the insurance systems and cultural differences (e.g. religion, family role, work/job), could differ in other countries. To conclude, the generalizability of the data is sufficient, but may not be applicable to all nationalities and cultures.

The created overview containing all factors that could determine prosthesis choice for persons with major unilateral ULD may help potential users and clinicians in the selection of a prosthesis. The overview provides a complete view of all factors that may matter when selecting a prosthesis. Considering the large number of factors the chances are conceivable that a person will not come up with all of these factors himself. Thus the overview could be used to make this large number of factors insightful for the potential users and for the clinicians, which will enable a well-informed shared-decision making process. The overview, provided by the clinicians, can be taken home by potential prosthesis users. They then can discuss it with relevant others and make their own decisions regarding factors that are important for their prosthesis selection. Involving people with a disability in the process of selecting the ‘right’ assistive device decreases device abandonment [17]. One way of using the framework in clinical practice could be to ask the potential prosthesis users to rank their five to ten most important factors. This list may facilitate the discussion between clinician and patient about the most preferred prosthetic components. Another approach to facilitate potential prosthesis users in decision-making could be by using an online decision aid for prosthesis users. Such tools are already available to assist in the choice of medical devices, such as blood glucose measurement instruments used by diabetic patients [53]. An online decision aid may help potential users to better understand different options and their impact, enhance involvement in decision-making and consider the importance of possible advantages and disadvantages [54]. The results of this study could provide the necessary information for the design of a decision aid for upper limb prosthesis users.

To conclude, the meta-synthesis of qualitative literature and focus group results of this study have provided an extensive overview of 86 factors that are important when selecting a prosthesis by persons with major unilateral ULD. The great number of factors confirms that preferences and needs with regard to upper limb prostheses vary greatly within prosthesis users. Therefore, it is important to take individual preferences into account in the selection of a prosthesis. A very limited number of qualitative studies on MHPs appeared to be available. To determine for whom the MHP is the best suitable prosthetic choice and for whom a less expensive option would be sufficient, further studies are needed. The created overview can be used to facilitate the conversation between users and clinicians on what really matters for that individual person. Ultimately this should lead to a better match between user and prosthesis, resulting in a decrease in upper limb prosthesis rejection rates and a higher cost-effectiveness of prosthesis-related health care.

## Supporting information

S1 Text Search terms BeHEMoTH (Behavior of Interest, Health context, Exclusions, and Models or Theories) used to search PubMed. (PDF)

S2 Text Search terms SPIDER (Sample, Phenomenon of Interest, Design, Evaluation, Research type) used to search PubMed. (PDF)

S3 Text Interview guide of the focus group. (PDF)

S1 Table Preliminary, prefinal and final frameworks with definitions and quotes. (PDF)

S2 Table List of all studies assessed for eligibility with full text screening. (PDF)

S3 Table PRISMA 2009 checklist. (PDF)

## References

[1] Ostlie K, Magnus P, Skjeldal OH, Garfelt B, Tambs K. Mental health and satisfaction with life among upper limb amputees: a Norwegian population-based survey comparing adult acquired major upper limb amputees with a control group. Disabil Rehabil. 2011;33: 1594–1607. 10.3109/09638288.2010.540293 21166612

[2] Sector M, Pezzin LE, Ephraim PL, MacKenzie EJ, Dillingham TR. Epidemiology of limb loss and congenital limb deficiency: A review of the literature. Arch Phys Med Rehabil. 2003;84: 747–761. 10.1016/s0003-9993(02)04932-8 12736892

[3] Dudkiewicz I, Gabrielov R, Seiv-Ner I, Zelig G, Heim M. Evaluation of prosthetic usage in upper limb amputees. Disabil Rehabil. 2004;26: 60–63. 10.1080/09638280410001645094 14660200

[4] Etter K, Borgia M, Resnik L. Prescription and repair rates of prosthetic limbs in the VA healthcare system: implications for national prosthetic parity. Disabil Rehabil Assist Technol. 2015;10: 493–500. 10.3109/17483107.2014.921246 24852068

[5] Edwards DS, Phillip RD, Bosanquet N, Bull AMJ, Clasper JC. What Is the Magnitude and Long-term Economic Cost of Care of the British Military Afghanistan Amputee Cohort? Clin Orthop Relat Res. 2015;473: 2848–2855. 10.1007/s11999-015-4250-9 26028596 PMC4523526

[6] Blough D, Hubbard S, McFarland L, Smith D, Gambel J, Reiber G. Prosthetic cost projections for servicemembers with major limb loss from Vietnam and OIF/OEF. J Rehabil Res Dev. 2010;47: 387–402. 10.1682/jrrd.2009.04.0037 20803406

[7] Zorginstituut Nederland / GIP. Totale kosten per gebruiker 2012–2016, hulpmiddelencategorie J01: Armprothesen [Internet]. [cited 8 Mar 2019]. Available: https://www.gipdatabank.nl/databank#/h//B_01-basis/tk_gebr/J01

[8] Biddiss E, Chau T. Upper-limb prosthetics: critical factors in device abandonment. Am J Phys Med Rehabil. 2007;86: 977–987. 10.1097/PHM.0b013e3181587f6c 18090439

[9] Biddiss E, Beaton D, Chau T. Consumer design priorities for upper limb prosthetics. Disabil Rehabil Assist Technol. 2007;2: 346–357. 10.1080/17483100701714733 19263565

[10] Cordella F, Ciancio AL, Sacchetti R, Davalli A, Cutti AG, Guglielmelli E, et al Literature review on needs of upper limb prosthesis users. Front Neurosci. 2016;10: 1–14. 10.3389/fnins.2016.00001 27242413 PMC4864250

[11] Resnik L, Klinger S, Gill A, Ekerholm Biester S. Feminine identity and functional benefits are key factors in women’s decision making about upper limb prostheses: a case series. Disabil Rehabil Assist Technol. 2018; 1–15. 10.1080/17483107.2018.1467973 29741966

[12] Kyberd PJ, Wartenberg C, Sandsjö L, Jönsson S, Gow D, Frid J, et al Survey of Upper-Extremity Prosthesis Users in Sweden and the United Kingdom. Am Acad Orthotists Prosthetists. 2007;19: 34–54. 10.4324/9780203001790

[13] Schaffalitzky E, NiMhurchadha S, Gallagher P, Hofkamp S, MacLachlan M, Wegener ST. Identifying the values and preferences of prosthetic users: A case study series using the repertory grid technique. Prosthet Orthot Int. 2009;33: 157–166. 10.1080/03093640902855571 19367519

[14] Østlie K, Lesjø IM, Franklin RJ, Garfelt B, Skjeldal OH, Magnus P. Prosthesis rejection in acquired major upper-limb amputees: A population-based survey. Disabil Rehabil Assist Technol. 2012;7: 294–303. 10.3109/17483107.2011.635405 22112174

[15] Østlie K, Lesjø IM, Franklin RJ, Garfelt B, Skjeldal OH, Magnus P. Prosthesis use in adult acquired major upper-limb amputees: Patterns of wear, prosthetic skills and the actual use of prostheses in activities of daily life. Disabil Rehabil Assist Technol. 2012;7: 479–493. 10.3109/17483107.2011.653296 22315926

[16] Kistenberg RS. Prosthetic choices for people with leg and arm amputations. Phys Med Rehabil Clin N Am. 2014;25: 93–115. 10.1016/j.pmr.2013.10.001 24287242

[17] Johnston P, Currie LM, Drynan D, Stainton T, Jongbloed L. Getting it “right”: How collaborative relationships between people with disabilities and professionals can lead to the acquisition of needed assistive technology. Disabil Rehabil Assist Technol. 2014;9: 421–431. 10.3109/17483107.2014.900574 24649888

[18] Stiggelbout AM, Van Der Weijden T, De Wit MPT, Frosch D, Légaré F, Montori VM, et al Shared decision making: Really putting patients at the centre of healthcare. BMJ. 2012;344: 1–6. 10.1136/bmj.e256 22286508

[19] Schweitzer W, Thali MJ, Egger D. Case-study of a user-driven prosthetic arm design: Bionic hand versus customized body-powered technology in a highly demanding work environment. J NeuroEngineering Rehabil. 2018;15 10.1186/s12984-017-0340-0 29298708 PMC5751817

[20] Davis C, St. Onge M. Myoelectric and Body-Powered Upper-Limb Prostheses: The Users’ Perspective. J Prosthetics Orthot. 2018; P30–P34. 10.1097/JPO.0000000000000155

[21] Van der Horst H, Hoogsteyns M. Disability, family and technical aids: A study of how disabling/enabling experiences come about in hybrid family relations. Disabil Soc. 2014;29: 821–833. 10.1080/09687599.2013.844102

[22] Zheng JY, Kalpakjian C, Larraga-Martinez M, Chestek CA, Gates DH. Priorities for the design and control of upper limb prostheses: A focus group study. Disabil Health J. 2019; 10.1016/j.dhjo.2019.03.009 30952491

[23] Luchetti M, Cutti AG, Verni G, Sacchetti R, Rossi N. Impact of Michelangelo prosthetic hand: Findings from a crossover longitudinal study. J Rehabil Res Dev. 2015;52: 605–618. 10.1682/JRRD.2014.11.0283 26437448

[24] Benz HL, Yao J, Rose L, Olgac O, Kreutz K, Saha A, et al Upper Extremity Prosthesis User Perspectives on Unmet Needs and Innovative Technology. Conf Proc IEEE Eng Med Biol Soc. 2016;25: 289–313. 10.1007/s11065-015-9294-9 PMC5508653 28268333

[25] Murray CD, Forshaw MJ. The experience of amputation and prosthesis use for adults: A metasynthesis. Disabil Rehabil. 2013;35: 1133–1142. 10.3109/09638288.2012.723790 23033871

[26] Carroll C, Booth A, Leaviss J, Rick J. “best fit” framework synthesis: Refining the method. BMC Med Res Methodol. 2013;13: 1 10.1186/1471-2288-13-1 23497061 PMC3618126

[27] Carroll C, Booth A, Cooper K. A worked example of “best fit” framework synthesis: A systematic review of views concerning the taking of some potential chemopreventive agents. BMC Med Res Methodol. 2011;11: 29 10.1186/1471-2288-11-29 21410933 PMC3068987

[28] Tong A, Flemming K, McInnes E, Oliver S, Craig J. Enhancing transparency in reporting the synthesis of qualitative research: ENTREQ. BMC Med Res Methodol. 2012;12: 1 10.1186/1471-2288-12-1 23185978 PMC3552766

[29] Booth A, Carroll C. Systematic searching for theory to inform systematic reviews: Is it feasible? Is it desirable? Health Info Libr J. 2015;32: 220–235. 10.1111/hir.12108 26095232

[30] Biddiss E, Chau T. The roles of predisposing characteristics, established need, and enabling resources on upper extremity prosthesis use and abandonment. Disabil Rehabil Assist Technol. 2007;2: 71–84. 10.1080/17483100601138959 19263542

[31] Nimhurchadha S, Gallagher P, Maclachlan M, Wegener ST. Identifying successful outcomes and important factors to consider in upper limb amputation rehabilitation: an international web-based Delphi survey. Disabil Rehabil. 2013;35: 1726–1733. 10.3109/09638288.2012.751138 23350754

[32] Biddiss EA, Chau TT. Multivariate prediction of upper limb prosthesis acceptance or rejection. Disabil Rehabil Assist Technol. 2008;3: 181–192. 10.1080/17483100701869826 19238719

[33] Cooke A, Smith D, Booth A. Beyond PICO. Qual Health Res. 2012;22: 1435–1443. 10.1177/1049732312452938 22829486

[34] Zeng X, Zhang Y, Kwong JSW, Zhang C, Li S, Sun F, et al The methodological quality assessment tools for preclinical and clinical studies, systematic review and meta-analysis, and clinical practice guideline: A systematic review. J Evid Based Med. 2015;8: 2–10. 10.1111/jebm.12141 25594108

[35] Hannes K, Lockwood C, Pearson A. A comparative analysis of three online appraisal instruments’ ability to assess validity in qualitative research. Qual Health Res. 2010;20: 1736–1743. 10.1177/1049732310378656 20671302

[36] Critical Appraisal Skills Programme. In: CASP (qualitative checklist) [Internet]. 2018 [cited 4 Apr 2019]. Available: https://casp-uk.net/casp-tools-checklists/

[37] Sandelowski M, Barroso J. Classifying the findings in qualitative studies. Qual Health Res. 2003;13: 905–923. 10.1177/1049732303253488 14502957

[38] Tong A, Sainsbury P, Craig J. Consolidated criteria for reporting qualitative research: A 32-item checklist for interviews and focus groups. Int J Qual Heal Care. 2018;19: 349–357. 10.1093/intqhc/mzm042 17872937

[39] Krueger AR, Casey AM. Focus Groups: A Practical Guide for Applied Research (3rd edition). Thousand Oaks, CA: Sage Publications 2000.

[40] Nagaraja VH, Bergmann JHM, Sen D, Thompson MS. Examining the needs of affordable upper limb prosthetic users in India: A questionnairebased survey. Technol Disabil. 2016;28: 101–110. 10.3233/TAD-160448

[41] Deijs M, Bongers RM, Ringeling-Van Leusen NDM, Van Der Sluis CK. Flexible and static wrist units in upper limb prosthesis users: Functionality scores, user satisfaction and compensatory movements. J Neuroeng Rehabil. 2016;13 10.1186/s12984-016-0130-0 26979272 PMC4791860

[42] Bouffard J, Vincent C, Boulianne E, Lajoie S, Mercier C. Interactions Between the Phantom Limb Sensations, Prosthesis Use, and Rehabilitation as Seen by Amputees and Health Professionals. J Prosthetics Orthot. 2012;24: 25–33. 10.1097/JPO.0b013e318240d171

[43] Waldera KE, Heckathorne CW, Parker M, Fatone S. Assessing the prosthetic needs of farmers and ranchers with amputations. Disabil Rehabil Assist Technol. 2013;8: 204–212. 10.3109/17483107.2012.699994 22779443

[44] Vasluian E, de Jong IGM, Janssen WGM, Poelma MJ, van Wijk I, Reinders-Messelink HA, et al Opinions of Youngsters with Congenital Below-Elbow Deficiency, and Those of Their Parents and Professionals Concerning Prosthetic Use and Rehabilitation Treatment. PLoS One. 2013;8 10.1371/journal.pone.0067101 23826203 PMC3691115

[45] Resnik L, Latlief G, Klinger SL, Sasson N, Walters LS. Do users want to receive a DEKA Arm and why? Overall findings from the Veterans Affairs Study to optimize the DEKA Arm. Prosthet Orthot Int. 2014;38: 456–466. 10.1177/0309364613506914 24286806

[46] McHugh ML. Interrater reliability: The kappa statistic. Biochem Medica. 2012; 22: 467–282. 10.11613/bm.2012.031 PMC3900052 23092060

[47] Saradjian A, Thompson AR, Datta D. The experience of men using an upper limb prosthesis following amputation: Positive coping and minimizing feeling different. Disabil Rehabil. 2008;30: 871–883. 10.1080/09638280701427386 17852212

[48] Widehammar C, Pettersson I, Janeslatt G, Hermansson L. The influence of environment: Experiences of users of myoelectric arm prosthesis—a qualitative study. Prosthet Orthot Int. 2018;42: 28–36. 10.1177/0309364617704801 28470129 PMC5808811

[49] Wijk U, Carlsson I. Forearm amputees’ views of prosthesis use and sensory feedback. J Hand Ther. 2015;28: 269–278. 10.1016/j.jht.2015.01.013 25990442

[50] Healy A, Farmer S, Eddison N, Allcock J, Perry T, Pandyan A, et al A scoping literature review of studies assessing effectiveness and cost-effectiveness of prosthetic and orthotic interventions. Disabil Rehabil Assist Technol. 2019;0: 1–7. 10.1080/17483107.2018.1523953 30652522

[51] Carey SL, Lura DJ, Jason Highsmith M. Differences in myoelectric and body-powered upper-limb prostheses: Systematic literature review. J Rehabil Res Dev. 2015;52: 247–262. 10.1682/JRRD.2014.08.0192 26230500

[52] Grypdonck MHF. Qualitative health research in the era of evidence-based practice. Qual Health Res. 2006;16: 1371–1385. 10.1177/1049732306294089 17079799

[53] Ekelmans N. Meer zorg op maat met online Keuzehulp Bloedglucosemeters. Ned Tijdschr voor Diabetol. 2018;16: 31–32. 10.1007/s12467-018-0015-x

[54] Stacey D, Légaré F, Lewis K, Barry MJ, Bennett CL, Eden KB, et al Decision aids for people facing health treatment or screening decisions. Cochrane Database Syst Rev. 2017 10.1002/14651858.CD001431.pub5 28402085 PMC6478132

