# Supplementary material for: User-relevant factors determining prosthesis choice in persons with major unilateral upper limb defects: A meta-synthesis of qualitative literature and focus group results
Source: PLoS One. 2020 Jun 30;15(6):e0234342. doi: 10.1371/journal.pone.0234342 (PMC7326229; doi:10.1371/journal.pone.0234342)
Supplement: S1 Text — (PDF) [file pone.0234342.s001.pdf]

S1 Text. Search terms BeHEMoTH (Behavior of Interest, Health context, Exclusions, and Models or Theories) used to search PubMed.

Below the used search string for PubMed is shown, this was adapted for use in the other bibliographic databases.

((Choice* [tiab] OR chos* [tiab] OR decision* [tiab] OR decide [tiab] OR consider* [tiab] OR select* [tiab] OR pick*[tiab]) AND (upper limb* [tiab] OR hand* [tiab] OR arm* [tiab] OR forearm* [tiab] OR transradial* [tiab] OR transhumeral* [tiab] OR forequar* [tiab] OR wrist disarticul* [tiab] OR elbow disarticul* [tiab] OR shoulder disartricul* [tiab]) AND prosthe* [tiab])) AND (Home* [tiab] OR daily lif* [tiab] OR daily liv* [tiab]) AND (Model* [tiab] OR theor* [tiab] OR framework* [tiab] OR concept* [tiab]) NOT (Non-theoretical model* [tiab] OR technical model* [tiab]OR statistical model* [tiab] OR economic model* [tiab])
