# Supplementary material for: User-relevant factors determining prosthesis choice in persons with major unilateral upper limb defects: A meta-synthesis of qualitative literature and focus group results
Source: PLoS One. 2020 Jun 30;15(6):e0234342. doi: 10.1371/journal.pone.0234342 (PMC7326229; doi:10.1371/journal.pone.0234342)
Supplement: S2 Text — (PDF) [file pone.0234342.s002.pdf]

S2 Text. Search terms SPIDER (Sample, Phenomenon of Interest, Design, Evaluation, Research type) used to search PubMed.

Below the used search strings for PubMed are shown, these were adapted for use in the other bibliographic databases.

Search 1:

((upper limb prosthe* [tiab] OR hand prosthe* [tiab] OR arm prosthe* [tiab] OR forearm prosthe* [tiab] OR transradial prosthe* [tiab] OR transhumeral prosthe* [tiab] OR forequarter prosthe* [tiab] OR upper limb amput* [tiab] OR hand amput* [tiab] OR arm amput* [tiab] OR forearm amput* [tiab] OR transradial amput* [tiab] OR transhumeral amput* [tiab] OR forequarter amput* [tiab] OR wrist disarticul* [tiab] OR elbow disarticul* [tiab] OR shoulder disartricul* [tiab])
OR ("Artificial Limbs"[Mesh] AND (upper limb* [tiab] OR hand* [tiab] OR arm* [tiab] OR forearm* [tiab] OR transradial* [tiab] OR transhumeral* [tiab] OR forequar* [tiab] OR wrist disarticul* [tiab] OR elbow disarticul* [tiab] OR shoulder disartricul* [tiab]))) AND (focus group* [tiab] OR questionnair* [tiab] OR survey* [tiab] OR interview* [tiab] OR opinion* [tiab] OR experien* [tiab] OR view* [tiab] OR concern* [tiab] OR motivat* [tiab] OR value* [tiab] OR desir* [tiab] OR perspectiv* [tiab] OR wish* [tiab] OR expectation* [tiab] OR OR attitud* [tiab] OR feel* [tiab] OR "Patient Satisfaction"[Mesh] OR useful* [tiab] OR satisf* [tiab] OR functional* [tiab] OR usage* [tiab] OR wear* [tiab] OR advantage* [tiab] OR disadvantage* [tiab] OR embodiment* [tiab] OR rejection rate* [tiab] OR cosmetic* [tiab] OR esthetic* [tiab] OR utilit* [tiab] OR comfort* [tiab] OR requirement* [tiab] OR need* [tiab] OR "Quality of Life"[Mesh] OR quality of life* [tiab] OR "Cost-Benefit Analysis"[Mesh] OR Accept* [tiab] OR reject* [tiab] OR value based health care [tiab] OR effectiv* [tiab] OR efficien* [tiab] OR preference* [tiab] OR econom* [tiab] OR cost* [tiab] OR OPUS* [tiab] OR TAPES* [tiab] OR QUEST* [tiab] OR qualitativ* [tiab] OR narrativ* [tiab] OR mixed method* [tiab] OR case stud* [tiab]) NOT (transplant* [tiab] OR pediatr* [tiab] OR 3D* [tiab])

Search 2:

(congenital [tiab] AND (upper limb* [tiab] OR hand* [tiab] OR arm* [tiab] OR forearm* [tiab] OR transradial* [tiab] OR transhumeral* [tiab] OR forequar* [tiab] OR transversal reduct* [tiab] OR wrist disarticul* [tiab] OR elbow disarticul* [tiab] OR shoulder disartricul* [tiab]) AND (defic* [tiab] OR defec* [tiab] OR abnormal* [tiab])) AND (focus group* [tiab] OR questionnair* [tiab] OR survey* [tiab] OR interview* [tiab] OR opinion* [tiab] OR experien* [tiab] OR view* [tiab] OR concern* [tiab] OR motivat* [tiab] OR value* [tiab] OR desir* [tiab] OR perspectiv* [tiab] OR wish* [tiab] OR expectation* [tiab] OR OR attitud* [tiab] OR feel* [tiab] OR "Patient Satisfaction"[Mesh] OR useful* [tiab] OR satisf* [tiab] OR functional* [tiab] OR usage* [tiab] OR wear* [tiab] OR advantage* [tiab] OR disadvantage* [tiab] OR embodiment* [tiab] OR rejection rate* [tiab] OR cosmetic* [tiab] OR esthetic* [tiab] OR utilit* [tiab] OR comfort* [tiab] OR requirement* [tiab] OR need* [tiab] OR "Quality of Life"[Mesh] OR quality of life* [tiab] OR "Cost-Benefit Analysis"[Mesh] OR Accept* [tiab] OR reject* [tiab] OR value based health care [tiab] OR effectiv* [tiab] OR efficien* [tiab] OR preference* [tiab] OR econom* [tiab] OR cost* [tiab] OR OPUS* [tiab] OR TAPES* [tiab] OR QUEST* [tiab] OR qualitativ* [tiab] OR narrativ* [tiab] OR mixed method* [tiab] OR case stud* [tiab]) NOT (transplant* [tiab] OR pediatr* [tiab] OR 3D* [tiab])
