# Supplementary material for: User-relevant factors determining prosthesis choice in persons with major unilateral upper limb defects: A meta-synthesis of qualitative literature and focus group results
Source: PLoS One. 2020 Jun 30;15(6):e0234342. doi: 10.1371/journal.pone.0234342 (PMC7326229; doi:10.1371/journal.pone.0234342)
Supplement: S3 Text — (PDF) [file pone.0234342.s003.pdf]

S3 Text. Interview guide of the focus group.

Time schedule and questions for participants of the focus group.

**Before start of focus group**	- Reception with coffee and tea;<br>- Participants signed the informed consent form
**Introduction (± 15 minutes)**	- A brief introduction of the subject and purposes of the meeting and explanation of the general rules of the focus group meeting (i.e. role moderator, respect for each others opinion, no need for consensus, etc.)
**Question 1 (± 15 minutes)**	- Which matters/factors influenced your prosthesis choice, or the choice to not use a prosthesis?
**Question 2 (± 15 minutes)**	- After you chose a prosthesis (or chose to not use a prosthesis), which matters/factors were important for you when using a prosthesis?
**Question 3 (± 30 minutes)**	- Participants were shown the themes and subthemes of the prefinal framework in a consecutive order, presented on large posters. Participants were asked to reflect on each theme following these questions:<br>1. Do you understand the mentioned theme and all subthemes? If necessary additional explanations were given.<br>2. Does this theme contain any subthemes that are not important to you? If yes, please explain?<br>3. Do you miss any subthemes of importance within this theme? If yes, please explain?
**End of focus group**	- Summary;<br>- Participants filled out a questionnaire with socio-demographic data (age, level of limb loss, origin of limb loss, current prosthesis, job);<br>- Participants were thanked for their participation.
