# Supplementary material for: User-relevant factors determining prosthesis choice in persons with major unilateral upper limb defects: A meta-synthesis of qualitative literature and focus group results
Source: PLoS One. 2020 Jun 30;15(6):e0234342. doi: 10.1371/journal.pone.0234342 (PMC7326229; doi:10.1371/journal.pone.0234342)
Supplement: S1 Table — (PDF) [file pone.0234342.s004.pdf]

S1 Table. Preliminary, prefinal and final frameworks with definitions and quotes.

Legend: PL, preliminary framework; PF, prefinal framework; F, final framework; MHP, multi-grip myoelectric hand prostheses; SHP, standard myoelectric hand prosthesis (with only one grip function); ULD = upper limb defect.

**Theme**	**Subtheme**	**Definition**	**Framework**			**Quote**
			**PL**	**PF**	**F**	
Physical	Gender	Gender of a person.	✓	✓	✓	‘She felt it was not designed for a “petite” female such as herself.’ *– Text from included study with quote of a DEKA-arm user* [11].
	Origin of limb loss	Cause of limb loss, for example: congenital limb deficiency, trauma, malignancy, etc.	✓	✓	✓	‘I don't know what I'm missing so I'm content.’ - Q*uote of a person with a congenital limb defect* [22].
	Hand dominance	Loss of dominant or non-dominant side of upper limb	✓			
	Level of limb loss	Portion of a limb that is still present.	✓	✓	✓	‘The higher up the arm the amputation is, the more difficult the fit and limited the function of a prosthesis.’ - *Text from commentary of a conventional myoelectric prosthesis user* [20].
	Phantom limb pain/sensations	Pain or sensations of the missing limb, like it is still attached.	✓	✓		‘In contrast [to body-powered prosthesis users], all myoelectric prosthesis users reported that their phantom limb sensations interacts with prosthesis use.’ *– Text from included study* [42].
	Phantom limb pain	Painful sensations of the missing limb, like it is still attached.			✓	‘…. And for me it [phantom limb pain] affected … wearing the prosthesis. That sometimes I just can’t wear it.’ *– Quote of participant from the focus group in this study.*
	Phantom limb sensations	Non-painful sensations of the missing limb, like it is still attached.			✓	‘… and ehh, that [prosthesis use] has nothing to do with the phantom limb, but I also think it is not a pain, absolutely not.’ *– Quote of participant from the focus group in this study.*
	General physical health	Overall physical well-being of a person including comorbidities and fitness level.	✓			
	Level of education	Level of highest degree of education achieved.	✓			
	Overuse symptoms	Pain symptoms due to overuse of for instance neck, back or unaffected arm. This also includes the prevention of these symptoms.		✓	✓	‘It’s [overuse complaints] worse these periods without the prosthesis, for example when it gets too tight during summer, and I get heat rash and I can’t wear the prosthesis to the same extent. Then I feel immediately it’s getting worse. I get a lot of relief with the prosthesis.’ *– Quote of a prosthesis user, unknown which type* [49].
	Skin irritation	Skin irritation, redness or inflammation of the stump skin due to prosthesis use.		✓	✓	‘The harness system was often cited as the cause of skin irritation and upper body pain leading to discomfort and disuse of the prosthesis…’ *– Text from included study* [9].
	Physical effort needed to	Physical effort that is needed to control the prosthesis.	✓	✓	✓	‘I love the body-powered system. However, I’m completely exhausted and in a lot of pain after a few hours. I attribute that specifically to the harness system.’ *– Quote of a body-*

	control					*powered prosthesis user* [9].
Activities and participation	Performing family/social role	Fulfilling social and family functions and needs (e.g. spouse, patent, breadwinner, friend).	✓			
	Performing family role	Fulfilling family functions and needs (e.g. spouse, parent, breadwinner).		✓	✓	'When the children were small, when both were very young, then in the beginning I didn't have the prosthesis because I felt that I was afraid of pinching them…' – *Quote of a prosthesis user, unknown which type* [49].
	Leisure activities	Hobbies and sports.	✓	✓	✓	'I've done various things...I've done archery, football, crown green bowls, ten pin bowling, darts tennis...There are ways to do different things. It might take you a couple of goes to do it, but you'll find out that you can do it.' – *Quote of a prosthesis user, unknown which type* [47].
	Self-care	Take care of yourself (e.g. brushing teeth, dressing, tying shoes)	✓	✓	✓	'… Another thing I discovered is that you can tie your shoe grasping laces between fingers… It improves the level of life that we do with prostheses.' - *Quote of a current MHP-user, with experience in using an SHP* [23].
	Household	Performing household activities (e.g. hanging laundry, vacuuming, cooking).	✓	✓	✓	'You do a lot more, without doubts. Even daily, at home, I live alone so . . . in washing dishes, for example, this [the Michelangelo prosthetic hand] is better.' – *Quote of a current MHP-user, with experience in using an SHP* [23].
	Transport	Moving yourself by car, bike or other transport methods.	✓	✓	✓	'I drive an ordinary car, five gears, handbrake, everything for a normal, able-bodied person.' – *Quote of prosthesis user, unknown which type* [47].
	Work/school	Activities related to school and/or work.	✓	✓	✓	'I couldn't go to work and not put it on 'cos I wouldn't be able to do my job.' – *Quote of a prosthesis user, unknown which type* [47].
	Grabbing, picking up and holding objects	Grabbing, picking up and holding objects, for instance: using a wheelbarrow, carrying (heavy) bags, or picking up a tray or drinking glass.		✓	✓	'Difficult to grasp and hold an object, Grasp of awkward shapes, Grasping of big/small objects, Slipping of objects, Slow' – *Text from table of included study about common complaints from patients in regard to their prosthesis* [40].
	Stabilize/ support objects	Stabilize/support objects, such as: keeping a paper in the right position when writing, keeping vegetables in the right place when cutting or holding a plank for screwing.		✓	✓	'… I'm very inefficient compared to what I used to be and the thing with holding the nail, putting it into the wall, that's an art of fine motor skill of holding it in the right spot, too.' – *Quote of prosthesis user, unknown which type* [22].
	Multi-tasking	Performing multiple activities simultaneously		✓	✓	'…walk and use arm at the same time…' – *Example of included study of an activities that persons preferred to do with their 'current device' instead of DEKA-arm* [45].
	Controlling electronic devices	Controlling devices such as the computer/laptop, mobile phone, or microwave.		✓	✓	'It was common to use it [the prosthesis] in productivity, at work and to do specific tasks such as using a computer or computer mouse, holding paper or plates, carrying things and hanging up laundry.' – *Text from included study* [49].
	Catching	Catching of objects, for instance a ball or keys being thrown			✓	'Well, I ehh, catching' - *Answer of a participant from the focus group in this study after we asked whether the shown theme was complete.*
Mental	Motivation	Motivation for prosthesis use.	✓	✓	✓	'No one will slow me down. I overdo it. I'm going to use my device'. – *Quotes of prosthesis users, unknown which types* [43].
	Attitude	Personal factors, character traits and principles that influences how someone	✓	✓	✓	'First, you must have a good attitude, otherwise, you won't use the device. We should do the best with what we've got and have faith.' – *Quote of prosthesis user, unknown which*

		thinks or acts in certain situations				*type* [43].
	Coping	Way someone deals with difficult situations (e.g. problem solving, handling reactions of others, etc.)	✓	✓	✓	'It depends a lot on what kind of person you are. For me, it was more that, yes, it has happened, let's go for it, as fast as we can, and then have another go, so to speak.' – *Quote of a myoelectric prosthesis user* [48].
	Expectations	Someone's expectations in regard of prosthesis use and whether these expectations are realistic.	✓	✓	✓	'Participants exhibited the importance of cognitive aspects in successful functional adjustment. This often involved a cognitive re-evaluation of functional abilities acknowledging the loss and in so doing adjusting expectations…' – *Text from included study* [47].
	Self-confidence	Self-confidence in regard to wearing a prosthesis.	✓	✓	✓	'There were some days at school when I was younger, when I couldn't have the prosthesis and it was terrible.' – *Quotes of prosthesis users, unknown which types* [49].
	Time since limb loss	Time in years since limb loss.		✓	✓	'I think in early years, there is a little bit of an embarrassment factor about it [having an amputation]...but I think as the years go on you get used to it, you just don't particularly notice it so much...I would imagine it still happens.' – *Quote of a prosthesis user, unknown which type* [47].
	Feeling disabled	Feeling that you are disabled with/without prosthesis		✓	✓	'Late adolescents, non-wearers, had negative feelings regarding the prosthesis. For them, the prosthesis was a statement about being disabled by highlighting the upper limb defect.' – *Text from included study* [44].
	Independence	Feeling that you are independent with/without prosthesis		✓	✓	'It's just that I like to be independent ...that's the kind of thing I like to put across that I am capable and ninety nine percent of the time I am able to function on my own.' – *Quote of prosthesis user, unknown which type* [47].
	Body embarrassment	Embarrassment for the own body with/without prosthesis		✓	✓	'I won't go swimming...my wife keeps telling me not to be so conscious but I can't help it.' – *Quote of a prosthesis user, unknown which type* [47].
	Need for prosthesis	Need for a prosthesis/ experiencing the added value of a prosthesis		✓	✓	'[As] a person with new limb loss, it's not so much what can't you do yet with your prosthesis. It's more what kind of activities have you done in the past that you would still like to continue doing.' – Quote of participant *with acquired ULD, prosthesis type unknown* [22].
	Self-image	How someone looks at themselves in regard to abilities, appearance and personality		✓	✓	'I have customized the look of my forearm by covering it with Swarovski crystals, and my prosthesis is not only extremely functional, but also it is a reflection of my creativity and spirit.' – *Text from commentary of a SHP- user* [20].
	Autonomy	Freedom of someone to decide for which activities he/she want to use the prosthesis (including unintended use) and how to use it.		✓	✓	'I'm not using it to its full capability I know, but I am using it to mine...for what I want...I love driving and it allows me to still be able to drive because it allows me the freedom of not having to have an automatic car if I don't want.' – *Quote of a prosthesis user, unknown which type* [47].
	Embodiment	Feeling that the prosthesis is part of the body/ feeling 'complete' with the prosthesis.		✓	✓	'The daily users talked in terms of "the prosthesis is a part of me" and "I can't function without it," which we interpreted as an embodiment of the prosthesis. The non-daily users all said "the prosthesis never became a part of me" or "I never felt comfortable with it."' – *Text from included study with quotes of myoelectric prosthesis users* [48].
	Mental effort needed to control	Mental effort that is needed to control the prosthesis.		✓	✓	'It just takes a little bit more thought...even little things, you have to think a lot more about them before you set about things' – *Quote of a prosthesis user, unknown which type* [47].

	Curiosity	Having a desire to know how it would be to have a (particular) prosthesis.			✓	'How's a prosthesis? And can I do more?' - *Quote of participant from the focus group in this study.*
Social	Support from family/friends	Supportive network with family and friends.	✓	✓	✓	'Support seemed to have an impact on prosthesis use, as all daily users described how they had been supported by their families or other social networks to use the prosthesis…' – *Text from included study* [48].
	Peer support	Support from other persons with upper limb deficiencies.	✓	✓	✓	'So how does one choose? One of the best ways to become familiar with the benefits and limitations of the different devices is to speak to individuals who wear them.' – *Text from commentary of a SHP-user* [20].
	Reactions from public	Reactions in public about having a prosthesis/upper limb deficiency (e.g. staring, unwanted attention, questions).	✓	✓	✓	'I also always use my prosthesis when I'm with people I do not know, even indoors. That is, I don't want other people to stare at me and all that…' - *Quote of a myoelectric prosthesis user* [48].
	Culture	Cultural background of a person.	✓			
	Fitting in	Belonging to a certain group; be the same as others.		✓	✓	'Regardless of whether the myoelectric prosthesis was used, all participants expressed that they wanted to be like, look like, or act like everyone else. Similar to most people in society, no one wanted to stand out.' – *Text from included study* [48].
	Performing social role	Fulfilling social functions and needs (e.g. as a friend, set of duties related to occupational status, in community), not related to the family.		✓	✓	'I was at a buffet . . . and, a glass of wine . . . I held a stem glass [with the Michelangelo prosthetic hand]! . . . Before, I had to take a plate from the buffet and find a table to eat, not now! Now, I can also walk . . . I mean . . . the fact that I have to sit at a table, it was something that isolated me from others. . . . Instead, now it is normal . . . wonderful!' – *Quote of a current MHP-user, with experience in using an SHP* [23].
	Advertisement	The influence of promotion and advertisement about arm prostheses on the prosthesis choice.			✓	'…on some [television] channel a "super hand", that could do everything, was shown... And then I have the whole app full again: "have you seen it?...." Yes I have seen that a long time ago, because I already discovered that earlier, but, you know… there is a kind of pressure on it anyway…' - *Quote of participant from the focus group in this study.*
	Anonymity	Not wanting to stand out or be stared at.			✓	'… if I am just at home, I just take it [the prosthesis] off, but for me that was indeed very important, how the environment reacts, that they don't immediately look at you, you still want to be a little bit anonymous in the environment… At least, that was my motivation for the prosthesis' - *Quote of participant from the focus group in this study.*
	Giving support to family/friends	Take away the worries of family/friends			✓	'… I also think, maybe, because if you're born like this [with an ULD], ehh, giving support to family. Because I know my mother had a hard time with it…' - *Quote of participant from the focus group in this study.*
	Modelling	Having an exemplary function for others, for instance showing them how you deal with having a short arm.			✓	'… that you can trigger something to people [of which they can learn], ehm yes, without you had actually planned that.' - *Quote of participant from the focus group in this study.*
	Prejudices of others	For example, other people taking over your tasks because they think you cannot do them yourself, while in reality that does not have to be the case.			✓	'…But it is true, something visible is often seen as a disability by an outsider…' - *Quote of participant from the focus group of this study.*
	Pressure of others	Pressure that people exert to wear or not to wear a (particular) prosthesis.			✓	'….I did volunteer work with an old lady and every week she asked: "well, do you take a prosthesis?" And then I thought ehh… No… But she asked it so often that I thought, now I

						want to know what it is like [to have a prosthesis]' - *Quote of participant from the focus group of this study.*
Rehabilitation, costs and prosthetist services	Access to service	Having access to rehabilitation services (e.g. travel distance, physical access, making appointments, acceptability of services).	✓	✓	✓	'Some drove over 3 h one way, some traveled out of state, and some even traveled by air to see their prosthetist!' – *Text from included study* [43].
	Cost of prosthesis	Costs of purchasing prosthesis and reimbursements for this.	✓	✓	✓	'Some farmers had no medical insurance and purchased their prostheses out-of-pocket. Most farmers reported that they do not receive worker's compensation because they are small business owners.' – *Text from included study* [43].
	Cost of maintenance	Costs of maintenance prosthesis and reimbursements for this.	✓	✓	✓	'Insurance paid for the initial prosthesis, but the deductible is so high that I can't afford replacement or repairs, so I need to be really careful with this one.' - *Quote of prosthesis user, unknown which type* [9].
	Information services	Being informed about the possible prosthesis options with their pros and cons.	✓	✓	✓	'Having conversations with an experienced upper-limb clinician and also meeting others in similar situations will help narrow the choices.' - *Text from commentary of an SHP-user* [20].
	Prosthesis training	Efficacy and satisfaction of prosthesis training services.	✓	✓	✓	'I moved from a big city to a small town, and there wasn't the same support to learn how to use it there that I had all my life. It disappeared completely. It was a setback. I stopped using the prosthesis altogether because it became really weird. I didn't get any support in this, and I became a prosthesis opponent instead.' – *Quote of a non-daily myoelectric prosthesis user* [48].
	Follow-up	Follow-up services.	✓			
	Time investment	Time-investment for choosing and fitting a prosthesis, prosthesis training, maintenance, follow-up and travel time.	✓	✓	✓	'A few [prosthesis users] said they had developed proficiency over many years with their simpler device and did not want to take the time to develop equal proficiency with a more complicated prosthetic system.*' – Text from included study* [45].
	Professional prosthesis maintenance	Prosthesis maintenance that should be done by a professional (like the prosthetist or therapist).		✓	✓	'It [the prosthesis] is being repaired at the moment… yes, now I got an "exchange" elbow… mine [elbow of participants prosthesis] is sent, repaired and then I have to come back again [to the prosthetist], to put that old one [elbow of participants prosthesis] back in there again.' - *Quote of participant from the focus group of this study.*
	Expertise of guidance	Expertise of the professionals involved in the process of choosing a prosthesis.			✓	'Yes, and that [why someone needs a certain prosthesis] should be well-argued, and then my rehabilitation doctor wrote a complete letter [to the insurance company] why it was needed … that is why I think expertise and expertise of guidance is very important.' - *Quote of participant from the focus group of this study.*
	Own costs	Own costs (deductible) of prosthesis, maintenance and training.			✓	'… If you order it [new gloves], you have to deduct it from your own risk to reimburse… yes, that will cost you €250 again… if you have no further healthcare costs then that is a shame actually.' - *Quote of participant from the focus group of this study.*
	Procedure insurances	Procedure and needed 'paperwork' that is required to request reimbursements from the health insurer			✓	'…I had a cosmetic prosthesis, I thought I needed it very badly. Well, that became a huge mess about the costs. In the end, that thing only cost 900 euros, I couldn't afford it myself, but that doesn't go about anything in health care country so to speak…' - *Quote of participant from the focus group in this study.*
	Trial period	Short period in which someone can try out			✓	'I had three arms, that I could try [at home]…. to see how everything went, and then I

		one or more prostheses at home, before actually choosing a prosthesis				could go home for two weeks, I could practice…. Yes, I liked that' - *Quote of participant from the focus group in this study.*
	Availability of new developments	Time that it takes until a certain new development in hand prosthesis is commercially available			✓	'For example … a 3D image was made of my arm, subsequently it is not printed [3D printed]' - *Quote of participant from the focus group in this study.*
Prosthesis related factors	Grip strength	Grip strength of the prosthesis	✓	✓	✓	'All participants found that the simple technique of opening and closing the prosthetic hand was fully sufficient but wished that the grip was stronger.' – *Text from included study* [48].
	Wrist control	Ability of a prosthesis to move and/or control the wrist joint.	✓	✓	✓	'The flexible wrist enabled movements that were not possible before.' - *Quote of a myoelectric prosthesis user* [41].
	Moving multiple joints	Ability of a prosthesis to move and control multiple joints (e.g. elbow, wrist, thumb, fingers).	✓	✓	✓	'Without shoulder reach function, the prosthesis is more hindrance than help.' – *Quote of a myoelectric prosthesis user with a high level amputation* [9].
	Ease in controlling	Ease of controlling the prosthesis	✓	✓	✓	'I have problems with the fine manipulations. I may not match the signals correctly, and then it will be. When I take the milk carton it is just squeezed together.' – *Quote of a non-wearer who had used a myoelectric prosthesis* [49].
	Dexterity	Fine motor skills.	✓	✓	✓	'Difficulty with precise hand or finger movements was a common challenge, both during activities of daily living and when using computers or handheld electronics.' – *Text from included study* [24].
	Reliability	Reliability of prosthesis (e.g. unplanned movements, slipping of objects, failure/malfunction, get broken).	✓	✓	✓	'The old prosthesis [the conventional myoelectric prosthesis] is more reliable. . . . Certainly, it [the MHP] has more functions but there are a lot of problems that need to be addressed from a technical point of view.' – *Quote of a current MHP-user, with experience in using an SHP* [23].
	Appearance	The way that the prosthesis looks and someone looks while wearing the prosthesis.	✓			
	Life-like appearance	Whether the prosthesis looks like a real arm.		✓	✓	'I would want them [the designer] to know that it looking more realistic, like a regular arm, would be like one of the biggest things that they could do.' – *Quote of a prosthesis user, unknown which type* [22].
	Size	The size of the prosthesis hand.		✓	✓	'Considering cosmetics, it [the MHP] seems more awkward . . . bigger, compared to the other one [the SHP]. The other one is proportioned to my hand.' - *Quote of a current MHP-user, with experience in using an SHP* [23].
	Appearance in combination with clothing	Appearance with prosthesis under clothing (e.g. not being able to wear certain clothes and/or not wanting to wear certain clothes due to the prosthesis)		✓	✓	'…I feel it's important 'cos people don't want me on their wedding photographs with, well I don't think so anyway, with your jacket sleeve tucked in your pocket. It looks a lot better with your prosthesis on.' – *Quote of a prosthesis user, unknown which type* [47].
	Wearing comfort	Wearing comfort of the prosthesis and comfort attachment approaches.	✓	✓	✓	'…there's a number of things that go into the comfort of a prosthesis. Number one is the socket. Where the prosthesis touches your skin. But also for a cable operated system it's the strap system. And the fact that I'm able to go harness free now with a myoelectric…' – *Quote of a person who uses both a body-powered and a myoelectric prosthesis* [24].

	Durability	Lifetime of the prosthesis and the separate parts of the prosthesis (i.e. gloves, battery, fingers).	✓	✓	✓	'For some, durability was one of the reasons why they preferred a body-powered device over a myoelectric device; they did not think that increased dexterity and function were enough to make up for how easily their myoelectric prostheses were damaged.' – *Text from included study* [22].
	Donning/ doffing	Effort needed to put the prosthesis on and off.	✓	✓	✓	'I was late for work a couple of times because I didn't estimate how much more time I would need to put on my arm when I first started wearing it, in the very beginning. Like, normally I would put my arm on as I was walking out the door in 2 seconds…' - *Quote of a DEKA-arm user* [11].
	Noise	Noise the prosthesis makes during movements.	✓	✓	✓	'This prosthesis [an MHP] is noisier than the other one [an SHP]. If I walk around . . . I don't say that people's heads turn, but they may say "What's wrong with him?"' – *Quote of a current MHP-user, with experience in using an SHP* [23].
	Weight	Weight of the prosthesis.	✓	✓	✓	'It [the MHP] is heavier [compared to the SHP], but you do better.' – *Quote of a current MHP-user, with experience in using an SHP* [23].
	Heat/ sweating	Heat and sweating while wearing the prosthesis.	✓	✓	✓	'You can get so sweaty, of course, so you almost feel that it's slipping off because you're sweaty…' - *Quote of a myoelectric prosthesis user* [48].
	Cold	Conduction of cold through the prosthesis.		✓	✓	'After a while the prosthesis cools down and then, of course, the stump also gets cold and when it gets cold it doesn't work properly, to open and close. That isn't good.' – *Quote of a myoelectric prosthesis user* [49].
	Ease in cleaning	Ease of cleaning the prosthesis.		✓	✓	'Farmers in the focus group cited the ease of cleaning their mechanical upper-limb prostheses with hot, soapy water as one reason they never adopted myoelectric prostheses.' – *Text of included study* [43].
	Water/ dirt proof	Resistance of prosthesis to water and dirt.		✓	✓	'One farmer reported that the control cable on a body-powered upper-limb prosthesis got stuck because of dirt clogging the cable housing. Particulates can also interfere with any unsealed mechanisms of electric powered components in myoelectric upper-limb prostheses.' – *Text from included study* [43].
	Self-maintenance	Maintenance of prosthesis that is done by the user themselves.		✓	✓	'Others learned ways to adapt their prosthesis to withstand certain activities, for example, using tape to keep the prosthesis together and attached to the arm when weight-lifting.' – *Text of included study* [22].
	Functionality	Having a (clear) function		✓	✓	'I am not interested [in cosmetics]. . . . I am not interested in having a cosmetic solution, it is always a prosthesis. The important thing is that it works, because if it doesn't . . . as I told you, I cannot do anything without prosthesis. I need its functionality. . . . I don't care if it has a color or another color. . . . It must work.' – *Quote of a current MHP-user, with experience in using an SHP* [23].
	Speed of movements	Speed of movements with prosthesis		✓	✓	'I only really have open/close on my myoelectric, which I also have with the DEKA, so it's really the additional features … its faster, but then you miss out on the weight side and the look of it.' – *Quote of a DEKA-arm user, who also uses MHP* [11].
	Natural movements/ postures	Being able to make natural movements with the prosthesis and/or having a more natural posture with the prosthesis		✓		'I realized the major impact of wrist movement; I was grabbing more things and in a more natural way.' - *Quote of a myoelectric prosthesis user* [41].
	Natural	Being able to make natural movements			✓	'I do it [activity of daily living] in a more natural manner [with an MHP in comparison with

	movements	with the prosthesis				an SHP]. . . . The movements are normal movements. . . . I do not block the wrist because I can do like this [leans prosthetic hand on leg]. Now, the shoulder is in a normal position.' – *Quote of a current MHP-user, with experience in using an SHP* [23].
	Body balance	Having a more natural posture with the prosthesis, being more in balance/symmetric			✓	'I like it [the prosthesis] a lot, because I have more balance. Yesterday I received a compliment from my trainer, who said, you have a good balance now with that arm.' - *Quote of participant from the focus group in this study.*
	Prosthesis fit	Fit of the prosthesis and changes therein, for example slipping of the prosthesis during activities or a poor prosthesis fit as a result of gaining/losing weight.			✓	'Yes, that's awkward, if you ehh.. have gained weight, than you also grown here [at the stump] and then it doesn't fit anymore…' - *Quote of participant from the focus group in this study.*
	Accessories	Accessories and tools that can be attached to a prosthesis or can be used to ease specific activities (such as cutlery, sport specific prosthesis, or typing tools)			✓	'…I can disconnect the lowest part of the arm … I have an ehh, phone holder, an I-pad holder, all different items. And that's the reason I can still do my work.' - *Quote of participant from the focus group in this study.*
	Elbow control	Ability of a prosthesis to move and/or control the elbow joint.			✓	'I would like to add elbow [to subtheme wrist control]' - *Answer of a participant from the focus group in this study after we asked whether the shown theme was complete.*
	Transporting the prosthesis	Ability to (easily) carry the prosthesis with you when it is not worn, because the prosthesis is particularly used for specific activities.			✓	'… also the transport of the prosthesis, because I don't wear it all the time, but then I had it in my bag and then I stood at the cheese farmer… he was startled.' - *Quote of participant from the focus group in this study.*
	Usability	Ease of using a prosthesis			✓	'… does it [the prosthesis] add something or would it get in the way too much, that could also be the reason not to opt for a certain prosthesis.' - *Quote of participant from the focus group in this study.*
	Vulnerability/ robustness	Ease in which the prosthesis can be damaged from the outside.			✓	'Yes, it [the prosthesis] should be strong. Strong and robust.' - *Quote of participant from the focus group in this study.*
