# Supplementary material for: User-relevant factors determining prosthesis choice in persons with major unilateral upper limb defects: A meta-synthesis of qualitative literature and focus group results
Source: PLoS One. 2020 Jun 30;15(6):e0234342. doi: 10.1371/journal.pone.0234342 (PMC7326229; doi:10.1371/journal.pone.0234342)
Supplement: S2 Table — (PDF) [file pone.0234342.s005.pdf]

**S2 Table. List of all studies assessed for eligibility with full text screening.**

| Study | Included or excluded? | Reason for exclusion (if applicable) |
|---|---|---|
| Stephens-Fripp B, Jean Walker M, Goddard E, Alici G. A survey on what Australian's with upper limb difference want in a prosthesis: justification for using soft robotics and additive manufacturing for customized prosthetic hands. Disabil Rehabil Assist Technol. 2019;0: 1–8. doi:10.1080/17483107.2019.1580777 | Excluded. | Non-qualitative study. |
| Zheng JY, Kalpakjian C, Larraga-Martinez M, Chestek CA, Gates DH. Priorities for the design and control of upper limb prostheses: A focus group study. Disabil Health J. 2019; doi:10.1016/j.dhjo.2019.03.009 | Included, except text about 'invasive prosthetic interfaces'. | 'Invasive cortical interfaces' are not commercial available and therefore text about these interfaces were excluded. |
| Janssen EM, Benz HL, Tsai J-H, Bridges JF. Identifying and prioritizing concerns associated with prosthetic devices for use in a benefit-risk assessment: a mixed-methods approach. Expert Rev Med Devices. 2018;15: 385–398. doi:10.1080/17434440.2018.1470505 | Excluded. | Study is about osseointegration, which is not commercially available yet. |
| Widehammar C, Pettersson I, Janeslatt G, Hermansson L. The influence of environment: Experiences of users of myoelectric arm prosthesis—a qualitative study. Prosthet Orthot Int. 2018;42: 28–36. doi:10.1177/0309364617704801 | Included. | |
| Schweitzer W, Thali MJ, Egger D. Case-study of a user-driven prosthetic arm design: Bionic hand versus customized body-powered technology in a highly demanding work environment. J NeuroEngineering Rehabil. 2018;15. doi:10.1186/s12984-017-0340-0 | Included qualitative parts of study. | Excluded non-qualitative parts of study. |
| Resnik L, Klinger S, Gill A, Ekerholm Biester S. Feminine identity and functional benefits are key factors in women's decision making about upper limb prostheses: a case series. Disabil Rehabil Assist Technol. 2018; 1–15. doi:10.1080/17483107.2018.1467973 | Included. | |
| Resnik L, Acluche F, Lieberman Klinger S, Borgia M. Does the DEKA Arm substitute for or supplement conventional prostheses. Prosthet Orthot Int. 2018;42: 534–543. doi:10.1177/0309364617729924 | Excluded. | Non-qualitative study. |
| Davis C, St. Onge M. Myoelectric and Body-Powered Upper-Limb Prostheses: The Users' Perspective. J Prosthetics Orthot. 2018; P30–P34. doi:10.1097/JPO.0000000000000155 | Included part about participant with unilateral upper limb defect. | Excluded part about participant with bilateral upper limb defects. |
| Lankhorst IMF, Baars ECT, van Wijk I, Janssen WGM, Poelma MJ, van der Sluis CK. | Excluded. | Age participants < 18 years. |

| | | |
|---|---|---|
| Living with transversal upper limb reduction deficiency: limitations experienced by young adults during their transition to adulthood. Disabil Rehabil. 2017;39: 1623–1630. doi:10.1080/09638288.2016.1206632 | | |
| Resnik LJ, Borgia ML, Acluche F. Perceptions of satisfaction, usability and desirability of the DEKA Arm before and after a trial of home use. PLoS One. 2017;12. doi:10.1371/journal.pone.0178640 | Excluded. | Non-qualitative study. |
| Benz HL, Yao J, Rose L, Olgac O, Kreutz K, Saha A, et al. Upper Extremity Prosthesis User Perspectives on Unmet Needs and Innovative Technology. Conf Proc IEEE Eng Med Biol Soc. 2016;25: 289–313. doi:10.1007/s11065-015-9294-9.Functional | Included. | |
| Nagaraja VH, Bergmann JHM, Sen D, Thompson MS. Examining the needs of affordable upper limb prosthetic users in India: A questionnairebased survey. Technol Disabil. 2016;28: 101–110. doi:10.3233/TAD-160448 | Included part about open-ended questions of survey. | Excluded parts with closed questions of survey. |
| Deijs M, Bongers RM, Ringeling-Van Leusen NDM, Van Der Sluis CK. Flexible and static wrist units in upper limb prosthesis users: Functionality scores, user satisfaction and compensatory movements. J Neuroeng Rehabil. 2016;13. doi:10.1186/s12984-016-0130-0 | Included qualitative parts of study. | Excluded non-qualitative parts of study. |
| Whelan L, Wagner N. Analysis of Factors Influencing Outcomes of Full and Partial Hand Multi-articulating Prostheses. J Hand Ther. 2016;29: 363. doi:10.1016/j.jht.2014.08.015 | Excluded. | No full text retrievable. |
| Luchetti M, Cutti AG, Verni G, Sacchetti R, Rossi N. Impact of Michelangelo prosthetic hand: Findings from a crossover longitudinal study. J Rehabil Res Dev. 2015;52: 605–618. doi:10.1682/JRRD.2014.11.0283 | Included qualitative parts of study. | Excluded non-qualitative parts of study. |
| Wijk U, Carlsson I. Forearm amputees' views of prosthesis use and sensory feedback. J Hand Ther. 2015;28: 269–278. doi:10.1016/j.jht.2015.01.013 | Included. | |
| Abd Razak NA, Abu Osman NA, Kamyab M, Wan Abas WAB, Gholizadeh H. Satisfaction and problems experienced with wrist movements: comparison between a common body-powered prosthesis and a new biomechatronics prosthesis. Am J Phys Med Rehabil. 2014;93: 437–444. doi:10.1097/PHM.0b013e3182a51fc2 | Excluded. | Non-qualitative study. |
| Resnik L, Latlief G, Klinger SL, Sasson N, Walters LS. Do users want to receive a DEKA Arm and why? Overall findings from the Veterans Affairs Study to optimize the DEKA Arm. Prosthet Orthot Int. 2014;38: 456–466. doi:10.1177/0309364613506914 | Included parts about home use, in which DEKA-arm is compared with their current device. | Excluded parts of the study that were non-qualitative or were not about home use. |

| | | |
|---|---|---|
| Van der Horst H, Hoogsteyns M. Disability, family and technical aids: A study of how disabling/enabling experiences come about in hybrid family relations. Disabil Soc. 2014;29: 821–833. doi:10.1080/09687599.2013.844102 | Included parts about adult upper limb prosthesis users. | Excluded parts about experiences with prosthesis as a child (<18 years) and all parts that did not describe the experiences of upper limb prosthesis users. |
| Lewis S, Russold MF, Dietl H, Kaniusas E. Satisfaction of Prosthesis Users with Electrical Hand Prostheses and their Sugggested Improvements. Biomed Tech (Berl). 2013;58 Suppl 1. doi:10.1515/bmt-2013-4385 | Excluded. | Non-qualitative study. |
| Vasluian E, de Jong IGM, Janssen WGM, Poelma MJ, van Wijk I, Reinders-Messelink HA, et al. Opinions of Youngsters with Congenital Below-Elbow Deficiency, and Those of Their Parents and Professionals Concerning Prosthetic Use and Rehabilitation Treatment. PLoS One. 2013;8. doi:10.1371/journal.pone.0067101 | Included parts about late adolescents. | Excluded parts about participants early adolescents, children and healthcare professionals. |
| Waldera KE, Heckathorne CW, Parker M, Fatone S. Assessing the prosthetic needs of farmers and ranchers with amputations. Disabil Rehabil Assist Technol. 2013;8: 204–212. doi:10.3109/17483107.2012.699994 | Included parts about upper-limb prosthesis users. | Excluded parts about lower limb prosthesis-users. |
| Bouffard J, Vincent C, Boulianne E, Lajoie S, Mercier C. Interactions Between the Phantom Limb Sensations, Prosthesis Use, and Rehabilitation as Seen by Amputees and Health Professionals. J Prosthetics Orthot. 2012;24:25-33. doi: 10.1097/JPO.0b013e318240d171 | Included qualitative parts about upper-limb prosthesis users. | Excluded non-qualitative parts and parts about health care professionals. |
| Østlie K, Lesjø IM, Franklin RJ, Garfelt B, Skjeldal OH, Magnus P. Prosthesis use in adult acquired major upper-limb amputees: Patterns of wear, prosthetic skills and the actual use of prostheses in activities of daily life. Disabil Rehabil Assist Technol. 2012;7: 479–493. doi:10.3109/17483107.2011.653296 | Excluded. | Non-qualitative study. |
| Kyberd PJ, Hill W. Survey of upper limb prosthesis users in Sweden, the United Kingdom and Canada. Prosthet Orthot Int. 2011;35: 234–241. doi:10.1177/0309364611409099 | Excluded. | No results of open-ended questions presented, so no qualitative content. |
| Berke GM, Fergason J, Milani JR, Hattingh J, Mcdowell M, Nguyen V, et al. Comparison of satisfaction with current prosthetic care in veterans and servicemembers from vietnam and OIF/OEF conflicts with major traumatic limb loss. J Rehabil Res Dev. 2010;47: 361–372. doi:10.1682/JRRD.2009.12.0193 | Excluded. | Non-qualitative study. |
| Reiber GE, Mcfarland L V., Hubbard S, Maynard C, Blough DK, Gambel JM, et al. Servicemembers and veterans with major traumatic limb loss from vietnam war | Excluded. | Non-qualitative study. |

| | | |
|---|---|---|
| and OIF/OEF conflicts: Survey methods, participants, and summary findings. J Rehabil Res Dev. 2010;47: 275–297. doi:10.1682/JRRD.2010.01.0009 | | |
| Murray CD. Being like everybody else: The personal meanings of being a prosthesis user. Disabil Rehabil. 2009; doi:10.1080/09638280802240290 | Excluded. | Target population could not be distinguished from other participants. |
| Schaffalitzky E, NiMhurchadha S, Gallagher P, Hofkamp S, MacLachlan M, Wegener ST, et al. Identifying the values and preferences of prosthetic users: A case study series using the repertory grid technique. Prosthet Orthot Int. 2009;33: 157–166. doi:10.1080/03093640902855571 | Included parts about upper limb prosthesis users (case 3 & 4). | Excluded parts about lower limb prosthesis users (case 1 & 2). |
| Saradjian A, Thompson AR, Datta D. The experience of men using an upper limb prosthesis following amputation: Positive coping and minimizing feeling different. Disabil Rehabil. 2008;30: 871–883. doi:10.1080/09638280701427386 | Included. | |
| Biddiss E, Chau T. Upper-limb prosthetics: critical factors in device abandonment. Am J Phys Med Rehabil. 2007;86: 977–987. doi:10.1097/PHM.0b013e3181587f6c | Excluded. | Non-qualitative study. |
| Kyberd PJ, Wartenberg C, Sandsjö L, Jönsson S, Gow D, Frid J, et al. Survey of Upper-Extremity Prosthesis Users in Sweden and the United Kingdom. Am Acad Orthotists Prosthetists. 2007;19: 34–54. doi:10.4324/9780203001790 | Included part about open-ended questions of survey. | Excluded parts with closed questions of survey. |
| Biddiss E, Beaton D, Chau T. Consumer design priorities for upper limb prosthetics. Disabil Rehabil Assist Technol. 2007;2: 346–357. doi:10.1080/17483100701714733 | Included parts about open-ended questions of survey and adult participants (as far as this could be distinguished). | Excluded parts about closed questions of survey and parts about paediatric population. |
| Pylatiuk C, Schulz S, Doderlein L. Results of an internet survey of myoelectric prosthetic hand users. Prosthet Orthot Int. 2007;31: 362–370. doi:10.1080/03093640601061265 | Excluded. | Non-qualitative study. |
